# Direct and indirect measurement of physical activity in older adults: a systematic review of the literature

**DOI:** 10.1186/1479-5868-9-148

**Published:** 2012-12-18

**Authors:** Kristina Kowalski, Ryan Rhodes, Patti-Jean Naylor, Holly Tuokko, Stuart MacDonald

**Affiliations:** 1 School of Exercise Science, Physical and Health Education and Department of Psychology, University of Victoria, Victoria, B.C, Canada; 2 Behavioural Medicine Laboratory, Faculty of Education, University of Victoria, Victoria, B.C, Canada; 3 School of Exercise Science, Physical and Health Education, University of Victoria, Victoria, B.C, Canada; 4 Department of Psychology, University of Victoria, Victoria, B.C, Canada

**Keywords:** Older adults, Physical activity assessment, Direct measurement, Indirect measurement, Self-report, Questionnaires, Accelerometry, Pedometery, Heart rate monitoring, Indirect calorimetry, Doubly labeled water

## Abstract

**Background:**

Due to physiological and cognitive changes that occur with aging, accurate physical activity (PA) measurement in older adults represents a unique challenge. The primary purpose of this study was to systematically review measures of PA and their use and appropriateness with older adults. A secondary aim was to determine the level of agreement between PA measures in older adults.

**Methods:**

Literature was identified through electronic databases. Studies were eligible if they examined the correlation and/or agreement between at least 2 measures, either indirect and/or direct, of PA in older adults (> 65 years of age).

**Results:**

Thirty-six studies met eligibility criteria. The indirect and direct measures of PA across the studies differed widely in their ability to address the key dimensions (i.e., frequency, intensity, time, type) of PA in older adults. The average correlation between indirect and direct measures was moderate (r=0.38). The correlation between indirect and other indirect measures (r=0.29) was weak, while correlations between direct measures with other direct measures were high (real world: r= 0.84; controlled settings: r=0.92). Agreement was strongest between direct PA measures with other direct measures in both real world and laboratory settings. While a clear trend regarding the agreement for mean differences between other PA measures (i.e., direct with indirect, indirect with indirect) did not emerge, there were only a limited number of studies that reported comparable units.

**Conclusions:**

Despite the lack of a clear trend regarding the agreement between PA measures in older adults, the findings underscore the importance of valid, accurate and reliable measurement. To advance this field, researchers will need to approach the assessment of PA in older adults in a more standardized way (i.e., consistent reporting of results, consensus over cut-points and epoch lengths, using appropriate validation tools). Until then researchers should be cautious when choosing measures for PA that are appropriate for their research questions and when comparing PA levels across various studies.

## Background

Older adults represent one of the fastest growing segments of our population. Worldwide, the proportion of adults aged 65 years and over was about 8 percent (521 million) in 2011 and it is anticipated that they could account for about 11 percent (939 million) of the total population by 2030 [1]. At this rate, it is anticipated that in the near future, the number of older adults will outnumber children for the first time in history [2,3]. Not only is the proportion of older adults increasing, but the average life expectancy also continues to climb [2,3].

Current physical activity (PA) guidelines encourage older adults to engage in at least 150 minutes of moderate- to vigorous-intensity aerobic PA per week and to engage in muscle and bone strengthening activities at least 2 days per week [4,5]. Despite these recommendations and the many well-known benefits of PA, studies have also demonstrated that the vast majority of older adults are physically inactive and that the prevalence of inactivity increases with advancing age [6-8]. With the growth in the number and the life expectancy of older adults, the numerous risks (e.g., disability, chronic disease, reduced functional abilities, increased falls) [9-13] associated with the prevalence of inactivity in older age have the potential to be an enormous burden not only to the older adult, but also to society as a whole.

Interventions aimed at improving levels of PA (i.e., “any bodily movement produced by the skeletal muscles that results in energy expenditure”) p. 126, [14] in older adults can have far reaching impacts on the aging population. Developing and evaluating interventions to meet this aim requires reliable, valid, cost-effective, practical and unobtrusive means of measuring PA. However, measurement of PA, especially in older adults, is, unfortunately, fraught with challenges. For example, changes in cognitive abilities and memory may lead to difficulties understanding instructions on self-report measures and challenges recalling PA behaviours, especially over longer periods of recall. Aging and disability changes the metabolic costs of activities, so standard tables and equations used for determining energy expenditure of activities that have been developed on younger populations may be inappropriate for older adults [12,15-19]. Existing indirect and direct measures of PA differ in their intended purpose, appropriateness for different populations and ability to assess the key dimensions of PA (frequency, intensity, time, and type (FITT) in older adults.

### Indirect PA measurement

Indirect measures rely on self-report [15,20-22] and are practical, easy to administer to large groups, and cost- efficient. They are also generally well accepted, place relatively low burden on and interfere little with the usual habits of the individual. However, they are prone to either over or under-estimation due to inaccurate recall, social desirability and misinterpretation [15,18,23]. In addition, many existing indirect tools fail to measure the lower end of the PA continuum [24] and are susceptible to fluctuations in health status, medical conditions and medications, fatigue, pain, concentration and distractibility, changes in mood, depression, and anxiety, and problems with memory and cognition [12,19,25].

### Direct PA measurement

Direct measures of PA assess energy expenditure [15] or actual movement [26] and are generally considered more accurate, are not prone to response and recall biases [15,20,22,27] and are often used to validate indirect measures of PA [20,22]. However, typically direct measures are more expensive, intrusive, time-consuming, and place a higher degree of burden on both the participant and the researcher than indirect measures [20,22]. Also, individuals may alter their behavior because they know it is being measured [15]. Some measures (e.g., accelerometers, pedometers) provide very limited information about type of activity [19] and are not suitable for measuring certain types of PA (e.g., swimming, resistance exercise, upper body movements, cycling, complex movements; [15,21]). Although direct measures do not rely on selfreport, there is a subjective element in data analysis and interpretation (i.e., the researcher chooses epoch lengths, cut points/thresholds for intensity groupings).

### Study purpose

Recent systematic reviews have compared direct and indirect measures of PA in adult [22] and pediatric [20] populations and found low to moderate correlations and poor agreement between direct and indirect measures of PA. Although assessment of PA in older adults represents a unique challenge, to the best of the author’s knowledge, a similar review of PA measures in older adults has not been conducted. Thus, the primary purpose of this paper is to provide a systematic review and critique of direct and indirect measures of PA and their use and appropriateness with older adults. To reach this end, tools were evaluated on their ability to assess the key dimensions of PA in older adults. The association and agreement between indirect and direct PA measures were also examined. Secondary objectives of this paper were to determine the relationship and agreement between: a) indirect measures with other indirect measures; and b) direct measures with other direct measures of PA in older adults.

## Methods

### Search strategy and selection

Literature searches of direct and indirect PA measures in older adults were conducted using ISI Web of knowledge, AgeLine, PsychINFO, Medline, and SPORT Discus (See Additional file 1. Search Strategy). The search strategy was developed by two of the authors (KK and RR) and was based on systematic reviews comparing direct and indirect measures of PA in adult [22] and pediatric populations [20]. Combinations of the following key terms were used to search the above databases: PA level terms (PA, exercise); older adult terms (older adults, aging, aged, seniors, elders, elderly, 65 years and over); general measurement terms (measures, measurement, instruments, tools, tests, assessment, testing); indirect measurement terms (indirect, subjective, self-report, diaries, logs, questionnaires, surveys, interviews); and direct measurement terms: (direct, objective, physical, doubly labeled water, indirect/direct calorimetry, accelerometry, pedometry, heart rate monitoring, GPS, direct observation).

Selected studies were peer reviewed journal articles examining the agreement between at least two measures, either indirect or direct, of PA in adults over 65 years. Studies were excluded if they: (1) did not compare at least two measures of PA, (2) involved a target population that included any participants less than 65 years of age, (3) were not written in English, or (4) were dissertations or conference presentations. Eligible direct measures included pedometry, accelerometry, heart rate monitoring, direct and indirect calorimetry, doubly labeled water, and direct observation. Eligible indirect measures included questionnaires, surveys, interviews, and activity records/logs/diaries. Other report (i.e., having significant others report on the individual’s physical activity) was considered an ineligible measure due to the possible heterogeneity of reporters (e.g., spouse, personal trainer, sibling, children, caregiver).

### Screening

**Figure 1:**
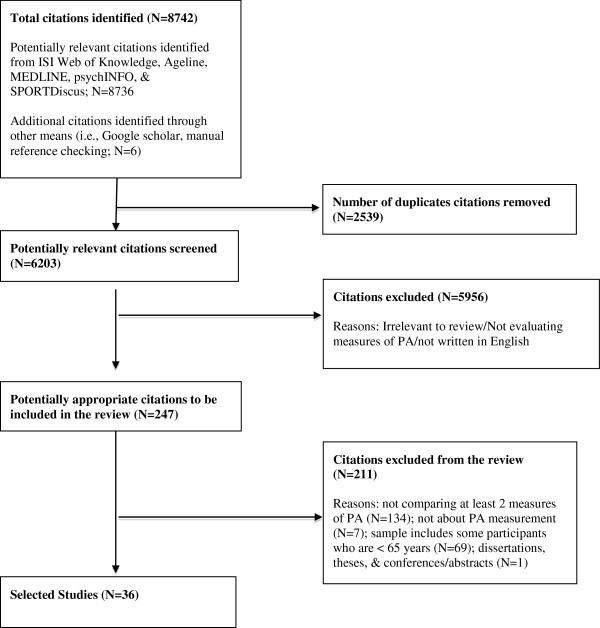
Screening Procedures.

The primary author initially screened identified studies based on the study title and abstract. Duplicates, articles that were not published in English, and irrelevant studies were manually removed. Potential studies were briefly scanned to see if they met eligibility criteria. Manual cross-referencing of bibliographies of the selected articles was also completed (See Figure 1. Screening Procedures).

### Quality assessment

The quality of studies was assessed using a recently developed checklist for evaluating the validity and suitability of existing activity and sedentary behavior instruments [28]. The checklist is an adaptation of the checklist created by Downs & Black [29]. This checklist includes additional criteria for questionnaire design and PA measurement and includes nine quality of reporting criteria, three external validity criteria, and ten internal validity criteria. Two reviewers, including one of the authors (KK), independently rated the quality of the individual papers and consensus was achieved through discussion.

### Data extraction and synthesis

Each study was reviewed and information about type of study design, sample characteristics, sample size (total, men, women), direct measures, units of measurement for direct measure, duration of direct measurement, indirect measures of PA, units of measurement for indirect measurement, length of recall, and time between each PA measure, correlations, and mean differences were extracted by the one author (KK) and a second author checked the data (RR). If level of agreement was not reported in the reviewed articles, but the units of measurement were comparable percent mean difference (indirect mean – direct mean/direct mean × 100) or absolute mean differences (direct mean – direct mean or indirect mean – indirect mean) were calculated. Units were converted to comparable units whenever possible. In many cases, units across the various measures of PA were not comparable; therefore, it was not possible to examine agreement between these PA instruments. As such, average correlations between: 1) direct and indirect measures; 2) indirect and indirect measures, and 3) direct and direct measures were also computed and adjusted/weighted by sample size. Since the correlation coefficient is not a linear function of the magnitude of the relation between two functions, and cannot simply be ‘averaged’, all correlations (Spearman rank coefficient, Pearson correlations coefficients) were first converted to Fisher’s Z, means and 95% confidence intervals (CIs) were computed, and then transformed back to correlation coefficients [30] using Comprehensive Meta-Analysis [31].

## Results

### Description of studies

A total of 8736 titles and abstracts were identified through online searches and were screened for inclusion. Of these records, 247 were examined more closely to determine their relevance after duplicates, non-English and clearly irrelevant articles (were not related to PA measurement) were eliminated. After eliminating additional studies that were not about PA measurement (N=7), did not examine at least 2 PA measures (N=134), did not involve the target population (N= 69), were dissertations, theses, and conferences/abstracts (N=1), 36 studies met inclusion criteria. Within these 36 studies, there were 21 studies that compared indirect with direct PA measures [16,26,32-50]. Eleven studies compared indirect measures with other indirect PA measures [16,32,33,37,45,47,48,51-54] and fourteen studies compared direct measures with other direct PA measures [38,41,43,55-65]. Eight of the latter studies only examined direct PA measures in a controlled or laboratory setting [56-59,62-65].

### Types of analyzes

Of the 21 studies examining the relationship between direct and indirect PA measures, nineteen reported correlational analyses [16,26,32-40,42,45-50] and six studies reported comparable data (e.g., kcal/day measured using a questionnaire and doubly labeled water [32,33,40,41,43,44]). Of the eleven studies examining indirect PA measures, eight studies reported correlational analyses [16,33,37,45,47,52-54] and six studies reported comparable data (e.g., min/day in PA measured using two different questionnaires; [16,33,46,52,54,55]). Of the 14 studies examining direct measures, 9 reported correational analyses [38,43,55,56,60-64] and 12 reported comparable data (e.g., step counts/day measured using a pedometer and accelerometer [38,41,43,55-59,61-65]).

### Description of study samples

Participants in eligible studies ranged from 65 to 99 years of age. Sample sizes ranged from eight [61] to more than 5000 [54]. The latter study reported on the results of a subset of questions from an annual US survey but the total sample of older adults was not reported. The majority of studies reported on combined samples of both genders, one reported on men only [32], four on women only [33,34,43,52] and in one study gender was unclear [61].

### Quality of studies

The quality of all included studies was assessed (n=36) using the tool described above. Scores on this tool ranged from 9 to 19 out of a maximum score of 22 points. The mean score on the tool was 14.7 (2.3). Of the 36 studies, 29 were considered modest quality (score of 6 to 16) and 7 were considered high quality (17 to 22). None were categorized as poor (0 to 5). Scores on the reporting criteria ranged from 3 to 9 (maximum 9 points) with a mean of 6.4 (1.3). The mean score on the external validity scale (out of 3) was 1.3 (0.5). The internal validity of the reviewed studies ranged from 2 to 10 with a mean score of 6.9 (1.6). For more information about the quality ratings see Additional file 2.

### Data synthesis

#### Brief overview of the indirect measures and their assessment of PA dimensions in older adults

Thirty-two different indirect measures were used to assess PA in the identified studies. These can be divided into two broad groups of PA questionnaires (i.e., self- or interview administered questionnaires/surveys) or activity logs (i.e., records kept for a specified timeframe) [21,23,66]. The most frequently used self-report measures were the Physical Activity Scale for the Elderly (PASE; n=8, including 1 translation [16,26,32,33,37,46,47,50]), the Commu-nity Healthy Activities Model Program for Seniors Activities Questionnaire for Older Adults (CHAMPS; n=4 [16,35,42,53]), and activity diaries/logs (n=5 [34,41,45,47,48]).

Self-report measures were classified by type according to the system described by Neilson and colleagues [67]. In this system, PA questionnaires (PAQs) that derive a score and contain less than 10 items are classified as global, PAQs that derive scores, activity duration, or estimate energy expenditure and contain 10–20 items are classified as recall questionnaires, and PAQs that derive an estimate of energy expenditure and contain more than 20 items are classified as quantitative. Of the 32 self-report measures identified in this review, 8 were classified as quantitative, 10 as recall, and 9 as global. Five additional questionnaires could not be located and were not evaluated (i.e., Physical Activity Index [33], Japan Arteriosclerosis Longitudinal Study Physical Activity Questionnaire [37], the Modified Dallaso [32], Older Adult Exercise Status Inventory [52], and a unspecified global PA item [47]).

As can be seen from Table 1, the self-report methods in this review varied in their ability to address the four PA dimensions. A majority of measures (21/27) asked about frequency of activities, but some only asked about the frequency of a limited number of the total list of activities evaluated in the measure. Although the scoring systems of thirteen measures involved assigning intensity codes or metabolic equivalents to activities endorsed by the older adult, only 6 measures required participants to rate the intensity of their activities (e.g., pace of walking, rate of exertion, rating scales). Most measures (22/27) evaluated at least one major type of PA (leisure, household, occupational), three categorized PA by intensity (e.g., light PA, moderate PA, vigorous PA), and two measures did not measure type of activity. Several of the tools (i.e., the CHAMPS, YPAS, PASE, Modified Baecke, the Phone FITT, the Physical Activity Questionnaire for the Elderly Japanese, the Questionnaire D’Activité Physique Saint Etienne, and the Older Adult Exercise Status Inventory, LASA Physical Activity Questionnaire) were designed specifically for older adults and address physical activities, including lower intensity activities, in which older adults are more likely to engage. All but seven measures asked about duration of activity, either in hours/week, hours or minutes/day or minutes/occasion. A substantial portion of the measures evaluated in this review asked about duration of activity per occasion, total volume across the day, or assessed duration on ratings scales.

**Table 1:** Basic characteristics and dimensions of physical activity assessed by self-report measures described in this review

**Name**^**a**^	**Type (method of administration**^**b**^**)**	**Length of Recall**	**Dimensions of PA (FITT)**
Minnesota Leisure Time Activity Questionnaire	Quantitative (Interview)	Last 12 months	**F:** months/year & average # times/month
			**I:** METs assigned to activities
			**T:** leisure time & home/household
			**T:** hours & min. of activity per occasion
Yale Physical Activity Survey	Quantitative (interview)	Typical week in last mo.	**F:** times/month of walking, vigorous intensity
			**I:** activities assigned intensity codes
			**T:** vigorous activity, leisurely walking, sitting, standing, stairs, activity across 4 seasons
			**T:** hours/week. Duration not rated per occasion.
Modified Baecke Questionnaire	Recall (Interview)	Past year	**F:** rating scales (e.g., never, sometimes, mostly, always)**,** months per year, stairs/day
			**I:** activities assigned intensity codes
			**T:** habitual physical activities (household, sport & other leisure)
			**T:** hours/week. Duration not rated per occasion.
College Alumni Questionnaire	Recall (interview/self)	Usual activity & past year. Past week	**F:** blocks/day, flights/day, times/year or times/week
			**I:** METs applied to activities, pace of walking, level of exertion of exercise
			**T:** leisure, walking, stair climbing, general vigorous, moderate, light activity, sitting activity, sleeping/reclining
			**T:** min/day; hours/day of activities on usual weekend & usual weekday
Seven Day Recall	Recall (interview)	Past 7 days	**F:** day/weeks, times/week (interviewer administered log of previous 7 days)
			**I:** Participant also asked to rate activities (moderate, hard, very hard). METs applied to activities
			**T:** sleep, leisure & occupational activities
			**T:** hours/day in moderate, hard, & very hard activities, hours of work, time spent sleeping
Lipid Research Clinics Questionnaire	Global (self)	Usual activity	**T:** leisure & occupational
Stanford Usual Activity Questionnaire	Global (interview)	Usual activity	**T:** leisure, walking, stairs
Physical Activity Scale forthe Elderly	Recall (interview or self)	Past 7 days	**F:** rating scale for leisure activities (never, seldom, sometimes, often)
			**T:** leisure, sedentary activity, walking, household & occupational
			**T:** rating scale for leisure activities (<hour, 1–2 hours, 2–4 hrs, >4 hrs). Hours of work per week.
Questionnaire d’Activité Physique Saint-Etienne	Quantitative (interview)	Typical week in past year	**F:** days/week, times/week (from typical timetable)
			**I:** METs assigned to activities – age & sex adjusted
			**T:** leisure, household, basic activities of daily living, travel including walking), & occupational
			**T:** min/week
Community Health Activity Model Program for Seniors Activities Questionnaire for Older Adults	Quantitative (interview/self)	Typical week in last month	**F:** times/week
			**I:** METS assigned to activities
			**T:** light, moderate, vigorous physical activity
			**T:** hours/week
Phone FITT	Recall (interview)	Typical week in last month	**F:** times/week, months/year
			**I:** Rating scale (breathing normally, slightly out of breath, too out of breath to carry out a conversation)
			**T:** household activities, recreational & conditioning, seasonal recreational
			**T:** time per occasion (1–15, 16–30,31-60 min, >1 hr
Zutphen Physical Activity Questionnaire	Recall (self)	Past wk. past mo. or usual	**F:** times/week, times/month
			**I:** Pace of walking. Intensity codes assigned to hobbies and sports;
			**T:** leisure time, walking, stairs, sleep, household, activities
			**T:** minutes/week
Physical Activity Questionnaire for Elderly Japanese	Recall (self)	Typical wk in past month	**F:** rating scale (never, seldom, sometimes, often)
			**I:** METs assigned to activities
			**T:** personal transportation, exercise/sports, household activities, occupational activities
			**T:** rating scale for hours per day(<1 hr, 1–2 hrs, 2-4 hrs, >4 hrs)
			**Other:**
Longitudinal Aging Study Amsterdam Physical Activity Questionnaire	Quantitative (interview)	Previous two weeks	**F:** times//2 weeks
			**I:** METS assigned to activities^c^
			**T:** walking outside, bicycling, gardening, light household activities, heavy household activities, & maximum of sports activities
			**T:** time per occasion (hours & min.)
Barcelona Health Interview Survey (Physical activity items)	Global (interview)	Previous week	**F:** times/week of bouts >20 minutes
			**T:** light, moderate, & vigorous physical activity
Exercise Induced Sweating (Godin & Shepard, 1985)	Global (interview/self)	Week	**F:** times/week
			**T:** regular activity long enough to work up a sweat
Behavioral Risk Factors Surveillance System	Recall (interview)	Past month	**F:** times/week or month
			**I:** METs assigned to activities
			**T:** household, occupational & leisure grouped
			**T:** hours & min per activity
Lifetime Physical Activity based on Godin et al. (1997)	Global (self)	Lifetime	FITT not evaluated. Rating of physical activity participation over entire life course on 5 point scale from “never been involved” to “always been involved”
National Health Interview Survey Heath & 1985 HD/DP supplement	Recall (interview)	Past 2 weeks	**F:** times/2weeks
			**I:** rated intensity of activity (small, , moderate, or large increase or no increase in heart rate or breathing)
			**T:** leisure time & occupational activity
			**T:** min per occasion of activity
24 hour recall	Quantitative (?)	24 hours	**F**: activities/5 min interval/day
			**I:** individually measured energy cost assigned to activities and total daily EE calculated
			**T**: sitting quietly, sitting actively, standing quietly, standing activity, walking, cycling
			**T**: min per activity
Job-related activity item	Global (self)	Current/not specified	**T:** Job related activity (physical work on job)
			**T:** rated on scale of great deal, a moderate amount, a little or none.
Daily activity item	Global (self)	Current/not specified	**T:** main daily activity (physical work)
			**T:** rated on scale of great deal, a moderate amount, a little or none.
Activity compared to peers item	Global (self)	Current/not specified	**T:** physical activity compared to peers on 3 point (more active, less active, or about as active) or 5 point scale (a lot more or a little more/a lot less or a little less)
Activity logs/diaries	Quantitative (self)	Varies, typically a week	**F:** times/day, times/week, which days/week
			**I:** ratings of perceived exertion. METs assigned to activities
			**T:** varies, all activities can be recorded
			**T:** depends on level of detail of log, time per occasion, hours per day.
Health Promoting Lifestyle Profile, exercise subscale	Global (self)	Present life (time frame not specified)	**F:** 4 point scale (Never, Sometimes, Often, Routinely)
			**T:** planned exercise, light to moderate PA, vigorous PA, leisure (recreational) PA, stretching, exercise during usual daily activities
Active Australia Survey	Recall (interview) –	Past week	**F:** times/week
			**T:** walking, vigorous gardening or heavy work around the yard, vigorous physical activity excluding gardening, household chores and yard work, moderate physical activity
			**T:** total time (hours and min) per activity categoriesin last week
International Physical Activity Questionnaire	Quantitative (self or interview). Note: short form also available (only 7 items)	Usual (long form) or past week (short form)	**F:** days/week
			**T:** vigorous PA, moderate PA, walking, sitting
			**T:** min and hours/day per activity^d^

^a.^Abbrevations: *FITT* frequency, intensity, time, type; *MET* metabolic equivalent.

^b.^The modified Dallaso, the Older Adult Exercise Status Inventory, the Physical Activity Index, or the Japan Arteriosclerosis Longitudinal Study Physical Activity Questionnaire, 24 hour recall (not described), and an unspecified global self-rated PA item are not summarized in this table because the authors were unable to obtain a copy of the questionnaires.

^c.^Types of physical activity questionnaires (PAQ) were classified using the same terminology as Neilson, Robson, Friendenreich, & Csizmadi [67], where global PAQs derive a score & contain <10 items, recall PAQs derive scores, activity duration or estimates of energy expenditure & contain 10–12 items and quantitative PAQs estimate EE and contain >20 items.

^d^.Stel et al., (2004) used a special scoring system scoring system for the Longitudinal Aging Study Amsterdam Physical Activity Questionnaire, rather than the usual scoring system. MET values were not assigned to activities in this study.

### Brief overview of the direct measures and their assessment of PA dimensions in older adults

Six different types of direct measures were employed, including accelerometers, pedometers, doubly labeled water, indirect calorimetry, heart rate monitoring, and direct observation. Detailed description of these instruments is outside of the scope of this review. For more information about these direct measures of PA instruments please refer existing review chapters and websites on PA measurement e.g., [15,21,23,66]. In the reviewed studies, accelerometry (21 studies) and pedometery (11 studies) were the most frequently used measures, while doubly labeled water (3 studies) and heart rate monitoring (2 studies) were the least frequently used direct measures. Accelerometry, pedometry, indirect calorimetry and heart rate monitor allow for quantifying intensity of exercise, but in very different ways (counts/min, oxygen consumption, changes in heart rate). As can be seen in Table 2, accelerometry, indirect calorimetry, heart rate monitoring and direct observation all provide some information about frequency and duration of PA, although to varying degrees.

**Table 2:** Basic characteristics and dimensions of physical activity assessed by direct measures described in this review

**Tool**	**Dimensions of Physical Activity**^**a**^
Accelerometry	**F:** bouts of continuous activity above a predetermined intensity threshold
	**I:** activity counts per unit time
	**T:** only activities where accelerations change. Activity of light, moderate, vigorous activity based on cut points.
	**T:** total volume of activity (min); time spent in activities above a predetermined intensity threshold level
	**Other**: energy expenditure can be calculated from calibration equation
Pedometry	**I:** Step counts per unit time.
	**T:** walking
Indirect Calorimetry	**F:** bouts of continuous activity above a predetermined intensity threshold
	**I:** average oxygen consumption
	**T:** activity of light, moderate, vigorous activity based on cut points.
	**T:** total volume of activity (min) times spent in activities above a predetermined intensity threshold level
	**Other:** energy expenditure can be calculated from oxygen consumption
Doubly labeled water	**FITT:** not evaluated
	**Other:** energy expenditure calculated from carbon dioxide production
Heart Rate Monitoring	**F:** bouts of activities/unit time greater than predetermined thresholds
	**I:** beats per min, average heart rate per day or time interval
	**T:** activities of different intensities based on cut points
	**T:** Duration of time spent activities of different intensities based on cut points
	**Other:** Energy expenditure can be calculated from group or individual calibration curves
Direct Observation	**F:** level of detail varies depending on observation protocol
	**T:** level of detail varies depending on observation protocol
	**T:** level of detail varies depending on observation protocol
	**Other:** Environment of activity, presence of others, behavioral cues, barriers

^a.^Abbreviations: *FITT* frequency, intensity, type, & time.

Four of the six measures permit calculation of type of activity by intensity, but provide very little or no information about the major types of PA [19]. For example, accelerometers cannot capture information about activities where there is no change in acceleration or that involve water (e.g., swimming, upper body movements, cycling [15,21]). Direct observation allows for detailed accounts of type, time and intensity of PA and is highly time consuming and places a lot of burden on the assessor. Pedometry is limited to monitoring acceleration/deceleration in the vertical plane. Doubly labeled water permits measurement of energy expenditure, as do accelerometry, indirect calorimetry, and heart rate monitoring, but provides no information about frequency, intensity, type or duration of PA. In addition, direct observation provides a means of looking at important variables that influence PA behaviors, including presence of others, behavioral cues, and barriers to PA in older adults.

### Agreement between indirect and direct measures

The results of studies of direct and indirect PA measures containing comparable units (n=6) are summarized in Table 3. For more information about the key characteristics of these studies see Additional file 3. Three of these studies examined the agreement between energy expenditure obtained from self-report measures and from doubly labeled water. In these studies, daily energy expenditure from self report both under and overestimated energy expenditure from doubly labeled water with values ranging from −14% to 37% [32,41,44]. Likewise PA energy expenditure was both under and overestimated by self-report compared to doubly labeled water with values ranging from −39 to 11 percent [32]. In two of the studies, differences between heart rate monitoring and self-report PA measures ranged from −14 to 6% percent. Last, PA from self-report both underestimated and overestimated PA as measured by accelerometer in the final two studies with comparable units [33,40].

**Table 3:** Agreement between measures of physical activity with comparable units in free living conditions

						**Absolute Difference**			
**Author (Year) & Sample**	**Comparison**	**Measure 1**	**Mean (SD)**	**Measure 2**	**Mean (SD)**	1. Difference between means on each measure	2. Mean of differences	**% Agreement**	**Limits of Agreement**^**a**^
Ayabe (2008) part 2^b^. All older adults	Direct vs. Direct	Pedometer (LC; waist); steps·day^-1^	8449 (3790)	Pedometer (YM; waist) steps·day^-1^	6798 (3569)		1652(862)	n/a	Not reported
Ayabe (2008) Part 2. Active older adults	Direct vs. Direct	Pedometer (LC; waist); steps·day^-1^	10736 (3465)	Pedometer (YM; waist) steps·day^-1^	9017 (3125)		1719(883)	n/a	−46 to 3484
Ayabe (2008) Inactive older adults	Direct vs. Direct	Pedometer (waist); steps·day^-1^	5402(1071)	Pedometer (waist) steps·day^-1^	3840 (1116)		1562(1719)	n/a	−164 to3289
Bergman (2009) part 2. Overall sample	Direct vs. Direct	Pedometer (SW3; waist) steps·day^-1^	1587 (1057)	Pedometer (DW; ankle) steps·day^-1^	6420(3180)	4833		n/a	
Bergman (2009) part 2. Men	Direct vs. Direct	Pedometer (waist) steps·day^-1^	1180(420)	Pedometer (ankle)	6565(1634)	5385			
Bergman (2009) part 2. Women	Direct vs. Direct	Pedometer (waist) steps·day^-1^	1767(1221)	Pedometer (ankle) steps·day^-1^ steps·day^-1^	6356 (3762)	4589			
Bonnefoy (2001). Men	Indirect vs. Direct	7 Day Recall TEE kcal·day^-1^	2810.8 (618.4)	DLW TEE	2,535.1 (585.2)	276		+10.8*	−1075 to 1625 kcal·day^-1^
Bonnefoy (2001). Men	Indirect vs. Direct	QAPSE TEE kcal·day^-1^	2177.1 (153.2) kcal·day^-1^	DLW TEE	2,535.1 (585.2)	358		−14.1*	−1470 to 754 kcal·day^-1^
Bonnefoy (2001). Men	Indirect vs. Direct	MLTPAQ EEPA kcal·day-1	490.9 (204.3) kcal·day^-1^	DLW EEPA	803.6 (463) kcal·day^-1^	313		−38.9*	−1188 to 562 kcal·day^-1^
Bonnefoy (2001). Men	Indirect vs. Direct	YPAS EEPA kcal·day^-1^	893.8 (395.8) kcal·day^-1^	DLW EEPA	803.6 (463) kcal·day^-1^	90		+11.3	−464 to 645 kcal·day^-1^
Bonnefoy (2001). Men	Indirect vs. Direct	CAQ EEPA kcal·day^-1^	563.1(246.6) kcal·day^-1^	DLW EEPA	803.6 (463) kcal·day^-1^	240		−30.0*	−1076 to 596 kcal·day^-1^
Bonnefoy (2001). Men	Indirect vs. Indirect	7 Day Recall TEE ^b^	2810.8 (618.4) kcal·day^-1^	QAPSE TEE	2177.1 (153.2) kcal·day^-1^	634	Not reported	Not applicable	Unable to calculate because mean (and) difference between measures not provided
Bonnefoy (2001). Men	Indirect vs. Indirect	YPAS EEPA	893.8 (395.8) kcal·day^-1^	MLTPAQ EEPA	490.9 (204.3) kcal·day^-1^	403	Not reported	Not applicable	Unable to calculate because mean (and) difference between measures not provided
Bonnefoy (2001). Men	Indirect vs. Indirect	YPAS EEPA	893.8 (395.8) kcal·day^-^	CAQ EEPA	563.1 (246.6) kcal·day^-1^	331	Not reported	Not applicable	Unable to calculate because mean (and) difference between measures not provided
Bonnefoy (2001). Men	Indirect vs. Indirect	MLTPAQ EEPA	490.9 (204.3) kcal·day^-1^	CAQ EEPA	563.1 (246.6) kcal·day^-1^	72	Not reported	Not applicable	Unable to calculate because mean (SD) difference between measures not provided
Conn (2000)	Indirect vs. Direct	Exercise EE (kcal/day)	388.85 (425.11)	Accelerometer (kcal/day)	275.77 (138.10)	112.73	Not reported	+40.1%	Unable to calculate because mean (SD) difference not provided
Harada (2001). Mixed sample. Retirement home sample	Indirect vs. Indirect	YPAS EE kcal·week^-1^	2313 (2277) kcal·week^-1^	CHAMPS EE kcal·week^-1^	1548 (1767) kcal·week^-1^	765	Not reported	Not applicable	Unable to calculate because mean (SD) difference between measures not provided
Harada (2001). Mixed sample. Community Centre Sample	Indirect vs. Indirect	YPAS EE kcal·week^-1^	8125 (4125) kcal·week^-1^	CHAMPS EE kcal·week^-1^	3848 (2402) kcal·week^-1^	4641	Not reported		Unable to calculate because mean (SD) difference between measures not provided
Harris (2009). Men (2009)	Direct vs. Direct	Pedometer (SW-200) steps·day^-1^)	6991(3919)	Accelerometer (Actigraph) steps·day^-1^	6931(3656)	60			
Harris (2009)) – Total Sample	Direct vs. Direct	Pedometer (SW-200) steps·day^-1^	6712 (3526)	Accelerometer (Actigraph) steps·day^-1^	6668 (3404)	44			
Harris (2009) –Women	Direct vs. Direct	Pedometer (SW-200) steps·day^-1^)	6428 (3088)	Accelerometer (Actigraph) steps·day^-1^	6401 (3136)	27			
Morio (1997) Men	Direct vs. Indirect	Activity log DEE (MH.day)	12.7 (2.2)	Doubly labeled water (MJ/day)	12.8 (3.1)			−0.9 (11.8)%	−13.9 to 17.7%
Morio (1997)	Direct vs. Indirect	Activity log DEE (MH.day)	12.7 (2.2)	HR monitoring (MJ/day)	13.5 (2.7)			−5.9%	Unable to calculate because mean (SD) difference not provided
Morio (1997) Women	Direct vs. Indirect	Activity log DEE (MJ/day)	8.8 (1.2)	Doubly labeled water (MJ/day)	9.6(0.8)			−7.7 (12.7)%	−21.9 to 12.3%
Morio (1997) Women	Direct vs. Indirect	Activity log DEE (MJ/day)	8.8 (1.2)	HR monitoring (MJ/day)	10.2(1.5)			−13.7%	Unable to calculate because mean (SD) difference not provided
Morio (1997) Men	Direct vs. Direct	HR monitoring (MJ/day)	13.5 (2.7)	Doubly labeled water (MJ/day)	12.8 (3.1)			4.5 (14.4)%	−9.0 to 32.3%
Morio (1997) Women	Direct vs. Direct	HR monitoring (MJ/day)	10.2(1.5)	Doubly labeled water (MJ/day)	9.6(0.8)			5.9(8.8)	−4.5 to 16.2%
Resnick (2008) – Senior Study	Indirect vs. Indirect	6 month YPAS PA	26.4 (13.8) hrs·week^-1^	6 month CHAMPS PA	19.9(10.1) hrs·week^-1^	6.5			
Resnick (2008) – Senior Study	Indirect vs. Indirect	6 month YPAS EEPA	5317.2 (2812) kcal·week^-1^	6 month CHAMPS EEPA	2979.0 (1719.9) kcal·week^-1^	2338.2			
Resnick (2008) – Senior Study	Indirect vs. Indirect	6 month YPAS MPA	2.4(3.3) hrs·week^-1^	6 month CHAMPS MPA	5.7(5.5) hrs·week^-1^	3.3			
Resnick (2008) – Senior Study	Indirect vs. Indirect	6 month YPAS EEMPA	742.7 (1090) kcal·week^-1^	6 month CHAMPS EEMPA	1030(1199.7) kcal·week^-1^	287.3			
Resnick (2008) –HIP study	Indirect vs. Indirect	Baseline, 6 & 12 month YPAS PA	30.0(27.7) 22.4(25.6) 21.4(21.3)	Baseline, 6 & 12 month CHAMPS	8.3(7.7)l 8.9(8.2) 8.6(7.8) hrs·week^-1^	21.7; 13.5; 12.8			
Resnick (2008) –HIP study	Indirect vs. Indirect	Baseline, 6 & 12 month YPAS EEPA	6002.6 (7357.2); 4715.3 (7177.8); 4306.7 (6337.2) kcal·week^-1^	Baseline, 6 & 12 month CHAMPS EEPA	1451.2 (1418.2) 1445.9 (1324.8) 1474.0 (1426.0) kcal·week^-1^	4551.4 3269.4 2832.7			
Resnick (2008) –HIP study	Indirect vs. Indirect	Baseline, 6 & 12 month YPAS MPA	0.9(1.9); 2.3(3.6); 1.9(3.2) hrs·week^-1^	B Baseline, 6 & 12 month CHAMPS MPA	2.1(3.8); 1.9(3.0) 2.3(3.8) hrs·week^-1^	1.2; 0.4; 0.4			
Resnick (2008) –HIP study	Indirect vs. Indirect	Baseline, 6 & 12 month YPAS EEMPA	287.0 (569.2); 529.1 (1001.8); 662.0 (1143.8) kcal·week^-1^	Baseline, 6 & 12 month CHAMPS EEMPA	486.7(862.8); 409.1 (608.1) 493.3 (777.7) kcal·week^-1^	199.7; 120; 168.7			
Rutgers (1997)	Indirect vs. Direct	24 hour recall	8.6(0.9) MJ/day	TEE HRM (individual calibration curve)	8.8(3.5) MJ/day		0.2(3.0)	−2.27	−6.22 to 5.85
Rutgers (1997)	Indirect vs. Direct	24 hour recall	8.6(0.9) MJ/day	TEE HRM (group calibration curve)	8.1(5.2) MJ/day		0.5(4.9)	+6.2	−9.41 to 10.39
Rutgers (1997)	Direct vs. Direct	TEE HRM (group calibration curve)	8.1(5.2) MJ/day	TEE HRM (individual calibration curve)	8.8(3.5) MJ/day		0.6(5.1)	n/a	−9.35 to 10.7
Seale (2002) Men	Indirect vs. Direct	DLW TEE	12.43(1.63)	EE from PA (7 day recall) and weight	17.03(4.07)		4.60(3.64)	+37.4(30.2)	−2.68 to 11.88
Seale (2002) Men	Indirect vs. Direct	DLW TEE	12.43(1.63)	EE from PA (7 day recall) and BMR	13.69(2.99)		1.26(2.63)	+10.58(21.8)	−4 to 6.52
Seale (2002) Men	Indirect vs. Direct	DLW TEE	12.43 (1.63)	EE from PA (7 day recall) and RMR	13.69(3.23)		1.27(2.46)	+9.8(19.9)	−3.65 to 6.19
Seale (2002) Women	Indirect vs. Direct	DLW TEE	9.44(0.90)	EE from PA (7 day recall) and weight	12.86(3.41)		3.42(3.69)	+38.03(39.4)	−3.96 to 10.8
Seale (2002) Women	Indirect vs. Direct	DLW TEE	9.44(0.90)	EE from PA (7 day recall) and BMR	10.15(2.21)		0.71(2.59)	+9.01(27.4)	−4.47 to 5.89
Seale (2002) Women	Indirect vs. Direct	DLW TEE	9.44(0.90)	EE from PA (7 day recall) and RMR	9.51(2.4)		0.07(2.76)	+2.2(29.1)	−5.45 to 5.59
Stel (2004)	Indirect vs Indirect	LAPAQ total activity	93 (4,5) score	6 month CHAMPS PA	89 (3,6) score	4			
Stel (2004)	Indirect vs Indirect	LAPAQ walking	14 (3,30)	6 month CHAMPS EEPA	28(18,38)	14			
Stel (2004)	Indirect vs Indirect	LAPAQ bicycling	0(0,10)	6 month CHAMPS MPA	0(0,10)	0			
Stel (2004)	Indirect vs Indirect	LAPAQ gardening	0(0,2)	6 month CHAMPS EEMPA	0(0,6)	0			
Stel (2004)	Indirect vs Indirect	LAPAQ light household	42 (28,56)	7 day diary light household	35 (22,48)	7			
Stel (2004)	Indirect vs Indirect	LAPAQ heavy household	4 (0,13)	7 day diary heavy household	6 (0,16)	2			
Stel (2004)	Indirect vs Indirect	LAPAQ Sports	2(0,16)	7 day diary Sports	0(0,9)	2			
Washburn (1990)	Indirect vs Indirect	BRFSS Standing light and moderate work	141.6 (153.9) min·day^-1^	Diary Standing light and moderate work	210.9 (107.3) min·day^-1^	8.085 hr/week			
Washburn (1990)	Indirect vs Indirect	BRFSS Slow walking	50.2(80.4) min·day^-1^	Diary Slow walking	54.2(38.7) min·day^-1^	0.467 hours/wk			
Washburn (1990)	Indirect vs Indirect	BRFSS Moderate walking	22.6(28.1) min·day^-1^	Diary Moderate walking	19.7(22.6) min·day^-1^	0.338 hours/wk			
Washburn (1990)	Indirect vs Indirect	BRFSS Light sport and recreation	9.9(26.7) min·day^-1^	Diary Light sport and recreation	11.9(31.3) min·day^-1^	0.233 hours/week			
Washburn (1990)	Indirect vs Indirect	BRFSS Moderate Sport and Recreation	3.9(7.2) min·day^-1^	Diary Moderate Sport and Recreation	4.1(11.1) min·day^-1^	0.023 hours/week			
Washburn (1990)	Indirect vs Indirect	BRFSS Total	228.4 (199.2) min·day^-1^	Diary total	300.9 (109.2) min·day^-1^	8.45 hours/week			

^a.^Limits of agreement = mean different ± 2s, where s = standard deviation of the differences

^b.^Study also examined step counts in younger adults (N=17). Only results specific to the older adult sample (N=28) are presented.

## Association between direct and indirect measures

While only 6 studies contained comparable data between indirect and direct measures, many reported on the association between variables. The correlations between direct and indirect measures of total levels of PA, regardless of direct method employed, fell in the weak to high range (−0.02 to 0.79). When all studies regardless of sample (i.e., men only, women only, mixed gender samples) were considered, the average correlation between indirect and direct measurement of total levels of PA was moderate **(**r=0.38, 95% confidence interval (CI) = 0.36 to 0.40). The average correlation between direct and indirect measures of total PA in samples of men only (r = 0.39, 95% CI= 0.33 to 0.45), studies including mixed samples of men and women (r = 0.39, 95% CI = 0.37 to 0.42) were also moderate, while the average correlation in samples of women only was weak (r = 0.252, 95% CI of 0.19 to 0.31).

## Agreement between indirect measures

As can be seen in Table 3, agreement between indirect PA measures varied considerably across the PA constructs (e.g., time, energy expenditure) and measures. For more information about the key characteristics of these studies see Additional file 4. For the purposes of comparison, energy expenditure scores were all converted to kcal/week and duration scores were converted to hours/week. Across studies reviewed in Table 3, absolute differences in agreement between different indirect measures of energy expenditure from PA varied from as low as a difference of 504 kcal/week between the YPAS and College Alumni Questionnaire [32] to as high as a difference of 7931 kcal/week between the YPAS and CHAMPS [53]. In both studies comparing the YPAS to the CHAMPS, the YPAS produced higher estimates of energy expenditure and of time spent in physical activity [16,53]. In the reviewed studies, differences between measures of time spent in physical activity varied from 0.4 hours per day to 21.7 hours per week [48,53].

## Association between indirect measures

In contrast to the limited studies evaluating agreement between indirect PA measures, many studies looked at the association of indirect measures with other indirect measures. Correlations between indirect measures of total levels of PA were in the weak to high range (0.15 to 0.85). When all studies (r=0.29, 95% CI = 0.28 to 0.30), and studies including mixed samples of men and women (r = 0.28, 95% CI = 0.27 to 0.29) were considered average correlations between indirect measures of total PA were weak. When correlations in samples of women only were considered, the average correlation was moderate (r = 0.46, 95% CI = 0.41 to 0.50).

## Agreement between direct measures

The findings regarding agreement between direct measures are grouped into those that measured PA levels in the real world (n=5; Table 3) and those that measured PA in controlled or laboratory settings (n= 10; Table 4). For more information about the key characteristics of these studies see Additional file 5. Among the 2 studies examining real world patterns of PA using pedometers, differences in daily step count from pedometers worn simultaneously over 7 days varied considerably ranging from 1562 steps per day to 5385 steps per day [55,56]. In contrast, step count differences between pedometers and accelerometers worn simultaneously over 7 days were very similar ranging from 27 steps per day for women to 60 steps per day for men in another study [38]. Compared to group calibrated heart rate monitoring [43] and doubly labeled water [41], individually calibrated heart rate monitoring provided higher estimates of daily expenditure in older adults.

**Table 4:** Agreement between mkeasures of physical activity with comparable units in controlled laboratory settings

**Author (Year)**	**Participants and/or conditions**	**Measure1**	**Mean (SD)**	**Measure 2**	**Mean (SD)**	**Difference**	**% Agreement**^**a**^
Bergman (2009) Part 1	Total sample, men, women	Pedometer (SW3; waist) steps	444(182); 476(237); 435(170)	Observed steps	433(175); 467(237); 423(162)	−11; 9; 12	
	Total sample, men, women	Pedometer (DW; ankle) steps	225(135); 200(146) 232(136)	Observed steps	433(175); 423(162); 435(170)	−208; −223; −203	
Cyarto (2004)	Nursing home slow, normal; fast pace	Pedometer (DW-200) waist)		Observed steps			−73.9(34.8); 55.1(37.8);-46.3(38.1)
	Senior centre slow, normal fast pace	Pedometer (DW-200 waist)		Observed steps			−24.7(36.1); −13.3(23.6); −7.1(26.2)
Fehling (1999)	Total sample/treadmill test	EE from accelerometer (Caltrac)		EE from indirect calorimetry			10% to 52%
	Exercise group/step test	EE from accelerometer (Caltrac)		EE from indirect calorimetry			−19% to −28%
	Total sample /treadmill test	EE from accelerometer (Tritrac)		EE from Indirect calorimetry			−12% to −37%
	Exercise group/step test	EE from accelerometer (Tritrac)		EE from Indirect calorimetry			−58% to −60%
Grant (2008)	Treadmill at 0.67, 0.9, 1.12, 1.34, 1.56 m/s	Pedometer (SW-200)		Observed steps	437(56); 490(55); 532(47); 585(47); 624(43)	184.3; 132.7; 71.8; 31.2; 4.0	42.2; 27.1; 13.5; 5.3; <1
	Treadmill at 0.67, 0.9, 1.12, 1.34, 1.56 m/s	Pedometer (NL-2000)		Observed steps	437(56); 490(55); 532(47); 585(47); 624(43)	85.4; 4.8; 0; −0.9; −2.4	19.5; <1; 0; <1; <1
Leaf (1995)	Treadmill	EE (kcals) from Indirect Calorimetry	43.4(8.41)	EE (kcals) from acceleromtery (Caltrac)	42.6(10.4)	−0.805	−1.86
	Treadmill	EE (kcals) from Indirect Caloriemtery	43.4(8.41)	EE (kcals) from ACSM equation	38.2(8.7)	−5.17	−11.92%
Marsh (2007)	131 m walk test	Pedometer (Accusplit Eagle 120) steps	196.0(62.6)	Observed steps	218.9(29)	−22.8(53.9)	−10.3(25.4)
		Pedometer (NL-2000) steps	214.9(27.2)	Observed steps	218.9(29)	−4.0(5.8)	−1.7(2.5)
		Accelerometry (IDEEA pattern recognition)	213.2(29.7)	Observed steps	218.9(29)	−5.6(7.8)	−2.5(3.7)
Resnick (2001)	One min walk tests	Step counter (SAM) steps	43.9(9.4)	Observed steps	43.05	0.85	1.98
Storti (2007)	Total Sample	Pedometer (DW) steps		Observed steps			−13%
	Slow, middle, fast gait	Pedometer (DW) steps		Observed steps			−31.2;-12.7; −11.1
	Total Sample	Accelerometer (Actigraph)		Observed steps			−7.1%
	Slow, middle, fast gait	Accelerometer (Actigraph) steps		Observed steps			−19.1;-5.7; −0.7
	Total sample	Step counter (SAM) steps		Observed steps			+6.9%
	Slow, middle, fast gait	Step counter (SAM) steps		Observed steps			+6.5;+6.6; +2.8

^a^Abbreviations: *TEE* daily total energy expenditure, *DLW* doubly labeled water, *QAPSE* Questionnaire D’Activité Physique Saint Etienne, *YPAS* Yale Physical Activity Survey, *MLTPAQ* Minnesota Leisure Time Physical Activity Questionnaire, *CAQ* College Alumni Questionnaire, *CHAMPS* Community Health Activity Model Program for Seniors Activities Questionnaire for Older Adults, *PA* Physical Activity, *DEE* daily energy expenditure, *EEPA* Energy expenditure from physical activity, *EEMPA* Energy expenditure from moderate physical activity, *MPA* Moderate physical activity, *HRM* heart rate monitoring, *RMR* resting metabolic rate, *BMR* basal metabolic rate, *LAPAQ* Longitudinal Aging Study Amsterdam Physical Activity Questionnaire, *BRFSS* Behavioral Risk Factors Surveillance System.

Note: Neither Harris (2009) nor Hurtig-Wennloff reported the data necessary to be included in this table (i.e., means, standard deviations, and/or absolute difference, percent agreement, limits of agreement)

Percent agreement calculated when one of direct measures was considered the reference measure.

Among the measures looking at the agreement between direct measures in controlled situations (i.e., treadmill tests, step tests, walking fixed distances), the most common comparisons were between pedometers or accelerometer step counts and observed step counts (manually or camera recorded step counts). With self-paced walking, pedometers (Accusplit Eagle 120 mechanical pedometer, NL-2000 electronic pedometer, the Step Activity Monitor, YAMAX DigiWalker) generally underestimated observed step counts, with percent agreement varying from −13% to +2% [63-65]. Likewise, accelerometers tended to underestimate actual observed step counts with percent agreement ranging from −7 to −3% [59,63,65]. In studies where speed of walking was considered, accuracy of pedometers and accelerometers tended to decrease as walking speed decreased [57,59,65]. Speed of walking was manipulated either by changing treadmill speed, having participants walk at self-selected speeds (slow, normal, fast) during a set distance, or by dividing participants into groups based on their gait speeds. In particular, the ActivPal, an accelerometer, stood out because it measured total steps and steps per min with a high degree of accuracy (i.e., errors less than 1% for both treadmill walking and walking outside; [59]).

Two measures examined the agreement between indirect calorimetry and accelerometry in estimating energy expenditure during exercise (treadmill, stepping test) [58,62]. Accelerometers tended to underestimate expended energy with estimates ranging from −2 up to −60%. However, this was not a uniform finding; accelerometers both overestimated (10–52%) and underestimated (−12% to −60%) energy expenditure from indirect calorimetry in one study [58] and underestimated energy expenditure (−2%) in the other [62].

## Association between direct measures

In most cases, studies comparing direct PA measures examined agreement rather than correlation. Of the studies looking at real world PA behavior, three studies reported correlational analyses between direct methods of measuring PA (i.e., pedometry with pedometry, accelerometer with pedometry, individually calibrated heart rate monitoring with group calibrated heart rate monitoring) with correlations ranging from 0.37 to 0.97 (r= 0.84, 95% CI = 0.81 to 0.87) [38,43,55].

The remaining studies also involved mixed samples of men and women and compared direct measures of PA in a laboratory setting with correlations varying considerably from weak (r =−0.28) for steps counted by direct observation and pedometry [56] to strong (r= 0.98 and r=0.99) for steps counted by direct observation and pedometry [56,63] and steps counted by direct observation and accelerometry [64]. The average correlation between direct PA measures in a laboratory setting, regardless of direct measure employed, was high (r=0.92, 95% CI = 0.90 to 0.94).

## Discussion

Reliable and valid assessment of PA in older adults is an important area of research. The quality of existing studies examining measurement of physical activity in older adults was moderate. Although the quality of the articles published on this topic was generally moderate and none of the studies were of poor quality, only 7 of the 36 studies were classified as high quality. These findings are informative but they need to be considered with some caution due to quality limitations of the studies at present. Without higher quality studies, significant gaps in our knowledge and understanding of PA measurement in older adults will remain. Higher quality research is needed to get a clearer picture of patterns of PA, to design interventions to promote PA, and to monitor changes in patterns of PA in older adults. To do so, researchers need to select valid measurement tools, and use stronger more consistent research methodology and superior reporting of results. Although systematic reviews of direct and indirect PA measurement tools have been conducted in adult and pediatric populations [20,22], to the best of the authors’ knowledge, this represents the first comprehensive attempt to: 1) evaluate the ability of PA measures to assess the dimensions of PA, and 2) assess the association and agreement between PA measures (i.e., direct with indirect, indirect the direct, and direct with direct), specifically in older adult populations.

### Indirect measures

The indirect measures that were reviewed differed widely in their ability to address the key PA dimensions in older adults. While self-report measures, including the more detailed PA questionnaires and activity logs, can be an excellent source of information of the dimensions of PA (especially frequency, time, and type of activity) in older adults, key limitations with respect to their use in older adults were identified in the selected studies. For instance, the high prevalence of assigning metabolic equivalents to activities in the reviewed studies is problematic considering standard tables developed with younger populations tend to overestimate the intensity of PA in older adults populations [17]. Age neutral measures were sometimes used in the selected studies. These questionnaires tend not to include the types of activities in which older adults typically participate [47,48]. Walking is the most common activity in which older adults participate [64], so those measures that specifically address walking intensity are of use with this population. Older adults generally tend to participate in lower intensity exercise more often than moderate and vigorous PA [25] and their PA participation tends to be intermittent, sporadic or unstructured making its recall more challenging [15,16]. Measures that permit the assessment of whether activity occurs in short bouts of activity or a single occasion is an important detail about the frequency and duration of activity [17]. Few of the self-report questionnaires examined in the selected studies asked participants to rate their own perceived intensity of activities. Perceived intensity differs depending on a person’s age and fitness level. An important consideration is that older adults, especially inactive ones, may perceive activities typically classified as light intensity, as more demanding than younger, more fit individuals.

### Direct measures

Direct measures of PA are generally considered to be more valid measures of PA than indirect measures. Like the reviewed self-report measures, the direct measures in this review varied in their ability to capture the key dimensions of PA. In particular, accelerometry and pedometry, the most frequently used direct measures in this review, are limited in their ability to capture type of activity. The direct measures in the selected studies were generally limited to the assessment of type of activity by intensity. While this PA dimension is very useful for addressing questions related to dose response to PA, it provides very limited information about the patterns of activity of older adults. Accelerometry, indirect calorimetry, and heart rate monitoring allow for evaluation of bouts of continuous activity above a predetermined intensity threshold, as well as total time above predetermined thresholds. These tools can provide a picture of the shorter and sporadic forms of activity in which older adults may participate. However, accelerometry, the least invasive and time intensive of the three types of measurement, is known to be less accurate at detecting PA at lower intensities, so bouts of continuous low intensity activity may be missed. Doubly labeled water, accelerometry, indirect calorimetry, and heart rate monitoring provide estimates of energy expenditure, however, there is a debate in the literature about the appropriateness of the calculations to estimate energy expenditure, especially for older adults populations [17,24,68].

A major limitation identified in the studies of direct PA measures was the methodological inconsistencies across studies (epoch lengths, cut points). Decisions about cut-points for classifying intensity levels, as well as selection of epoch lengths varied considerably across studies of accelerometers in this review. Moreover, within a single PA measurement tool, such as accelerometry, PA can be quantified in very different ways. For instance, the reviewed studies varied considerably in epoch lengths and cut-points for determination of PA intensity classifications and provided limited rationale provided for their choices. Although work has been done examining epoch lengths on estimates of physical activity in children, little research has been conducted with adults and even less with older adults. As has been found in recent work with post-menopausal women, it seems appropriate that shorter epoch lengths (e.g., 10 seconds) will derive more accurate estimates of physical activity in populations of older adults than longer epoch lengths (e.g., 1 min); however, the same study also found that relations of physical activity to most health outcomes did not vary by epoch lengths [69]. Moreover, national PA surveys with adults (e.g., National Health and Nutrition Survey, the Canadian Health Measures Survey) have generally used 1 minute epoch lengths [70,71]. Some work has developed cut-points of PA classification specific to older adults [72]; however, cut points that are not age specific are often used in research with this population. Decisions regarding cut-points can have dramatic effects on data interpretation, and the resulting PA classification levels (i.e., over or under-estimation of minutes of moderate to vigorous PA [73] and relationships between PA and various outcomes (e.g., health, cognition) [74]. Future research will need to establish age specific cut-points for older adults. Direct PA measurement in older adults is also complicated by the prevalence of slower walking speeds and gait disorders [15]. As found in this review, motion sensors are less accurate at slower speeds and this is a likely issue with older adult populations. Several tools identified in this review have been designed for and/or are appropriate for individuals with varying gait patterns and walking speeds [56,59,63]. An additional problem with objective measures of PA in older adults is low compliance with measurement protocols (e.g., problems with memory, lacking the visual and manual dexterity to put the device on properly and to activate it, confusion with using unfamiliar new technology [12,25,75]). Many of the selected studies either did not examine or did not report on compliance levels with direct measurement protocols. Studies in this area should address this factor as valid and reliable PA measures are of limited utility if older adults will not comply with the measurement. Thus, although the reviewed tools provide useful information about the dimensions of PA in older adults, there are many issues specific to older adults that make assessment of PA a unique challenge. These issues require further examination.

### Agreement and association between measures

Additional objectives of this study were to assess the agreement and association between: 1) indirect and direct measures, 2) indirect and other indirect measures, and 3) direct and other direct measures. Unfortunately, a clear pattern regarding the agreement of measures in these three groups did not emerge. Inconsistency in the type of results reported and the lack of comparable data in studies comparing 1) indirect and direct measures and 2) indirect measures with other indirect measures precluded the evaluation of percent agreement (or absolute difference) in the majority of instances. Findings regarding agreement between direct measures with other direct measures were mixed, with some measures yielding high levels of agreement and others not. Most studies of agreement between direct measures in this review examined accelerometers, pedometers, and direct observation. The limited scope of current research examining agreement between PA measures makes it difficult to compare across studies and to generalize results.

Studies of older adults relied primarily on correlational analyses to compare and to validate measures of PA in older adults. Similar to the systematic reviews of Adamo and colleagues [20] and Prince and colleagues [22], weak to moderate correlations were generally found between indirect and direct measures of PA in older adult populations. Likewise, the strength of the association between indirect measures with other indirect measures was generally weak, while it was the associations between direct measures and other direct PA measures, regardless of setting (i.e., real world, laboratory setting) were high. As has been noted by others, correlation provides information about the strength of the relationship and does not reflect agreement [22,76]. We must be cautious in relying solely on correlation as justification for choosing one measure over another. Moderately or even highly correlated measures may be measuring entirely different PA constructs. The results from this review provide limited information about the agreement across PA measures and minimal information to help guide researchers in their choice of PA measure. Researchers are advised to use the Quality Assessment of Physical Activity Questionnaire (QAPAQ) Checklist [77] to help researchers in their choice of PA self-report measures [77].

### Take home message

Despite the lack of clear trend regarding the agreement of PA measures in older adults, the findings provide useful information about research needs in this important field. Consistent with papers on pediatric and adult population agreement and correlations between measures were weak to moderate. The measurement of PA is complex across all populations and as one can see from this review, the measurement of PA involves additional unique challenges in older adults. Not only does PA involve a number of separate dimensions, but it also is not a static behavior. We cannot assume that physical activities are performed at the same intensity across person or time. There is considerable inter-individual variability (e.g., difference in perceived intensities of exercise, types of activities engaged in, and in the costs of PA) and intra-individual variability (e.g., disease states, changes in activity levels due to changes in health or demands on time). Moreover, the accuracy of our instruments also contributes to the weak to moderate correlations and agreement between measures.

Choosing the appropriate tool to measure PA in older adults is also complex. PA levels and patterns are more a reflection of biological age than chronological age. However, as Shephard [24] points out an agreed upon method of determining biological age does not exist. In healthy, active older adults assessment methods appropriate for younger populations may be quite appropriate. Based on the complexity of PA and its measurement, we cannot really expect to adequately capture all of its dimensions with a single measure. The question is not as simple as which measure is most appropriate for older adults, but rather what combination of tools is the most appropriate. The choice in tools depends not only on the specific population of older adults, but the intended purpose of the evaluation.

### Future directions

With regards to the appropriateness of PA measures in older adults, qualitative work is needed to help gain a better understanding of how older adults feel about these methods of measurement and the level of burden that is being place on them. Moreover, qualitative work can be used to help design new measures that address PA constructs specific to older adults or to improve on existing measures. Further work is needed to develop standard metabolic cost tables and equations that are specific to older adults. Questionnaire items should also be carefully developed to address not only the types of activities in which older adults participate and the generally more sporadic nature of older adult’s activities, but should also allow older adult to report on their perceived intensity during activity to allow for exploration of this important aspect of their PA. With regards to direct PA measures, further work is needed to address methodological inconsistencies (cut-points, epoch lengths), especially in older adults where limited research has been conducted. Achieving consensus within the research community in this regard is an important research goal. Assessment of factors such as health status, medical conditions and medications, changes in mood, depression, and anxiety, and fatigue, pain, and concentration and distractibility [10,22,23] that may influence PA measurement is important. Continuing to assess the agreement between PA measures in older adults is another important research target. If measures are found to show high agreement on the PA construct of interest, than the briefer, more feasible methods can be selected. To advance this field, PA researchers need to approach the assessment of PA in older adults in a standardized way. We cannot sufficiently assess agreement between measures unless researchers report the required results, reduce methodological inconsistencies (e.g., lack of consensus regarding cut-points, epoch lengths) and choose appropriate tools against which to validate their measures of PA (i.e., must be evaluating the same construct).

## Conclusions

In conclusion, considerable work is needed to advance the field of PA measurement in older adults. There are significant gaps in our knowledge about agreement between PA measures in older adults. Researchers should be cautious when choosing measures for PA and in comparing PA levels across studies, especially when different tools are being used.

## Abbreviations

PA: Physical activity; GPS: Global positioning systems; CI: Confidence interval; PASE: Physical Activity Scale for the Elderly; CHAMPS: Community Healthy Activities Model Program for Seniors Activities Questionnaire for Older Adults; YPAS: The Yale Physical Activity Survey; PAQ: Physical activity questionnaires; FITT: Frequency, Intensity, Type, Time.

## Competing interests

The authors declare that they have no competing interests.

## Authors’ contributions

KK and RR conceived the paper and methods. KK participated in the coordination of the search, conducted the literature search, assessed study quality, analyzed and interpreted the results, and drafted the majority of the manuscript. RR also provided comments to the writing. All authors advised on the initial concept, edited and approved the final manuscript.

## Authors’ information

KK is a research assistant and PhD candidate in the School of Exercise Science, Physical and Health Education, and the Department of Psychology at the University of Victoria, Canada.

RR is a professor and Canadian Cancer Society Senior Scientist in the School of Exercise Science, Physical and Health Education, University of Victoria, Canada.

PJN is a Professor in the School of Exercise Science, Physical and Health Education, University of Victoria, Canada.

HT is the Director of the Centre on Aging and Professor in the Department of Psychology at the University of Victoria, Canada.

SM is a Professor in the Department of Psychology at the University of Victoria, Canada.

## Supplementary Material

Additional file 1 **Search strategy for ISI Web of Knowledge and EBSCOhost (October 2011). **Description: This file contains the search terms used in this review, the databases that were searched and the hit rates for search terms. Click here for file

Additional file 2 **Quality of studies using the tool developed by Hagstromer & Bowles[**[29]**].** Description: This file contains the results from the tool used to assess the quality of each study included in the review. Each study was assessed by series of questions listed below the table. Click here for file

Additional file 3 **Title: Characteristics of studies comparing direct and indirect measures of physical activity in older adults.** Description: This document contains a table in which the key details (First author, sample, age (mean (SD), age range), sample size, direct measure (units), indirect measure (units), measurement details (timing, cut-points, epoch lengths, tests, and correlations) of studies comparing direct and indirect measures of physical activity in older adults have been summarized. Click here for file

Additional file 4 **Characteristics of studies comparing indirect measures of physical activity in older adults.** Description: This document contains a table in which the key details (First author, sample, age (mean (SD), age range), sample size, direct measure (units), indirect measure (units), measurement details (timing, cut-points, epoch lengths, tests, and correlations) of studies comparing indirect measures of physical activity with other indirect measures of physical activity in older adults have been summarized. Click here for file

Additional file 5 **Characteristics of studies comparing direct measures of physical activity in older adults.** Description: This document contains a table in which the key details (First author, sample, age (mean (SD), age range), sample size, direct measure (units), indirect measure (units), measurement details (timing, cut-points, epoch lengths, tests, and correlations) of studies comparing direct measures of physical activity with other direct measures of physical activity in older adults have been summarized. Click here for file
