# Supplementary material for: Direct and indirect measurement of physical activity in older adults: a systematic review of the literature
Source: Int J Behav Nutr Phys Act. 2012 Dec 18;9:148. doi: 10.1186/1479-5868-9-148 (PMC3549726; doi:10.1186/1479-5868-9-148)
Supplement: Additional file 1 — Search strategy for ISI Web of Knowledge and EBSCOhost (October 2011). Description: This file contains the search terms used in this review, the databases that were searched and the hit rates for search terms. [file 1479-5868-9-148-S1.docx]

**Appendix 1. *Search Strategy for ISI Web of Knowledge and for EBSCOhost (October 2011)***

|  | **Search Terms** | **Results for ISI Web of Knowledge** | **Results for EBSCOhost** |
| --- | --- | --- | --- |
|  | “direct measure*” OR “direct assessment” OR “objective measure* OR “objective assessment” OR “indirect calorimetry” OR “direct calorimetry” OR “doubly labeled water” OR “accelerometer*” OR “pedometer*” OR “Global positioning systems” OR “GPS” OR “heart rate monitor*” | [101,938](http://apps.webofknowledge.com/summary.do?product=WOS&doc=1&qid=1&SID=2CnLCjlmnBL4o3AdF@o&search_mode=GeneralSearch) | 60,886 |
| 2. | “Indirect measure*” OR “indirect assessment” OR “subjective measure*” OR “subjective assessment” OR “self-report” OR “self report” OR diaries OR logs OR surveys OR questionnaires OR interview* | [1,099,838](http://apps.webofknowledge.com/summary.do?product=WOS&doc=1&qid=2&SID=2CnLCjlmnBL4o3AdF@o&search_mode=GeneralSearch) | 1,731,649 |
| 3. | measure* OR assess* or instrument* or tool* | [5,497,776](http://apps.webofknowledge.com/summary.do?product=WOS&doc=1&qid=3&SID=2CnLCjlmnBL4o3AdF@o&search_mode=GeneralSearch) | 4,692,568 |
| 4. | “Physical activity” OR exercise OR “physical fitness” | [269,160](http://apps.webofknowledge.com/summary.do?product=WOS&doc=1&qid=4&SID=2CnLCjlmnBL4o3AdF@o&search_mode=GeneralSearch) | 516,007 |
| 5. | “Older adults” OR seniors OR elder* OR “65 years of age and over | [150,376](http://apps.webofknowledge.com/summary.do?product=WOS&doc=1&qid=5&SID=2CnLCjlmnBL4o3AdF@o&search_mode=AdvancedSearch) | 40,1190 |
| 6.  7.  8.  9.  10.  11.  12.  13. | #4 and #3  #4 and #1  #4 and #2  #8 or #7 or #6  #8 or #7  #9 and #5  #10 and #5  #11 or #12 | [99,094](http://apps.webofknowledge.com/summary.do?product=WOS&doc=1&qid=6&SID=2CnLCjlmnBL4o3AdF@o&search_mode=CombineSearches)  [6,647](http://apps.webofknowledge.com/summary.do?product=WOS&doc=1&qid=7&SID=2CnLCjlmnBL4o3AdF@o&search_mode=CombineSearches)  [33,373](http://apps.webofknowledge.com/summary.do?product=WOS&doc=1&qid=8&SID=2CnLCjlmnBL4o3AdF@o&search_mode=CombineSearches)  [112,331](http://apps.webofknowledge.com/summary.do?product=WOS&doc=1&qid=9&SID=2CnLCjlmnBL4o3AdF@o&search_mode=CombineSearches)  [37,801](http://apps.webofknowledge.com/summary.do?product=WOS&doc=1&qid=10&SID=2CnLCjlmnBL4o3AdF@o&search_mode=CombineSearches)  [7,423](http://apps.webofknowledge.com/summary.do?product=WOS&doc=1&qid=11&SID=2CnLCjlmnBL4o3AdF@o&search_mode=CombineSearches)  [3,553](http://apps.webofknowledge.com/summary.do?product=WOS&doc=1&qid=12&SID=2CnLCjlmnBL4o3AdF@o&search_mode=CombineSearches)  [6,401](http://apps.webofknowledge.com/summary.do?product=WOS&doc=1&qid=16&SID=2CnLCjlmnBL4o3AdF@o&search_mode=CombineSearches) | 163,738  8,468  66,604  194,955  72,514  14,205  6,764  2,335 |
|  | **Total:** | 8736 | |
