# Supplementary material for: Direct and indirect measurement of physical activity in older adults: a systematic review of the literature
Source: Int J Behav Nutr Phys Act. 2012 Dec 18;9:148. doi: 10.1186/1479-5868-9-148 (PMC3549726; doi:10.1186/1479-5868-9-148)
Supplement: Additional file 2 — Quality of studies using the tool developed by Hagstromer & Bowles[[29]]. Description: This file contains the results from the tool used to assess the quality of each study included in the review. Each study was assessed by series of questions listed below the table. [file 1479-5868-9-148-S2.doc]

**Additional File 2. Quality of studies using the tool developed by Hagstromer & Bowles [29]**

| Author year) |  | Reporting | | | | | | | | |  | EV | | |  | IV | | | | | | | | |  | Total Score |
| --- | --- | --- | --- | --- | --- | --- | --- | --- | --- | --- | --- | --- | --- | --- | --- | --- | --- | --- | --- | --- | --- | --- | --- | --- | --- | --- |
|  |  | 1 | 2 | 3 | 4 | 5 | 6 | 7 | 8 | 9 |  | 1 | 2 | 3 |  | 1 | 2 | 3 | 4 | 5 | 6 | 7 | 8 | 9 |  |  |
| Ayabe (2010) |  | 1 | 1 | 0 | 0 | n/a | 1 | 0 | 1 | 1 |  | 0 | 0 | 1 |  | 1 | n/a | n/a | 1 | 0 | n/a | 1 | 1 | 0 |  | 14 |
| Bergman (2009) |  | 1 | 1 | 1 | 0 | n/a | 1 | 0 | 1 | 1 |  | 0 | 0 | 1 |  | 1 | n/a | n/a | 1 | 0 | n/a | 1 | 1 | 0 |  | 16 |
| Bonnefoy (2001) |  | 1 | 1 | 1 | 0 | 1 | 1 | 0 | 1 | 1 |  | 0 | 0 | 1 |  | 1 | 0 | 1 | 0 | 0 | 0 | 1 | 1 | 0 |  | 12 |
| Conn (2000) |  | 1 | 1 | 1 | 0 | 1 | 1 | 1 | 1 | 0 |  | 0 | 0 | 1 |  | 1 | 0 | 1 | 0 | 0 | 0 | 1 | 1 | 2 |  | 14 |
| Cyarto (2004) |  | 1 | 1 | 1 | 0 | n/a | 1 | 1 | 0 | 1 |  | 0 | 0 | 1 |  | n/a | n/a | n/a | 1 | n/a | n/a | 1 | 1 | 0 |  | 16 |
| Dinger (2004) |  | 0 | 1 | 1 | 0 | 1 | 1 | 1 | 1 | 0 |  | 0 | 0 | 1 |  | 1 | 0 | 1 | 1 | 1 | 1 | 1 | 1 | 2 |  | 16 |
| Dominguez-Berjon |  | 0 | 1 | 0 | 0 | 1 | 1 | 0 | 1 | 1 |  | 1 | 0 | 1 |  | 1 | 0 | n/a | 1 | 0 | 0 | 1 | 1 | 2 |  | 13 |
| Fehling (1999) |  | 1 | 1 | 0 | 0 | n/a | 0 | 0 | 1 | 0 |  | 0 | 0 | 1 |  | n/a | n/a | n/a | 0 | n/a | n/a | 1 | 1 | 0 |  | 12 |
| Gerdhem (2008)[[1]](#endnote-2) |  | 1 | 1 | 1 | 0 | 0 | 1 | 1 | 1 | 0 |  | 1 | 0 | 1 |  | 1 | 0 | 1 | 1 | 0 | 1 | 0 | 1 | 2 |  | 15 |
| Giles (2009) |  | 1 | 1 | 1 | 1 | 1 | 1 | 1 | 1 | 0 |  | 0 | 0 | 1 |  | 1 | 1 | 1 | 1 | 1 | 1 | 1 | 1 | 2 |  | 19 |
| Gill (2008) |  | 1 | 1 | 1 | 1 | 1 | 1 | 1 | 1 | 1 |  | 1 | 0 | 1 |  | 1 | 0 | 0 | 1 | 1 | 1 | 1 | 1 | 2 |  | 19 |
| Grant (2008) |  | 1 | 1 | 0 | 0 | n/a | 1 | 1 | 1 | 1 |  | 0 | 0 | 1 |  | n/a | n/a | n/a | 1 | n/a | n/a | 1 | 1 | 0 |  | 16 |
| Hagiwara (2008) |  | 0 | 1 | 1 | 1 | 1 | 0 | 0 | 0 | 0 |  | 1 | 0 | 1 |  | 0 | 0 | 0 | 0 | 0 | 1 | 1 | 1 | 2 |  | 11 |
| Harada (2001) |  | 1 | 1 | 1 | 1 | 1 | 0 | 1 | 0 | 0 |  | 0 | 0 | 1 |  | 1 | 0 | 0 | 1 | 1 | 1 | 1 | 1 | 2 |  | 15 |
| Harris (2009) |  | 1 | 1 | 1 | 0 | 1 | 0 | 1 | 0 | 1 |  | 1 | 1 | 1 |  | 1 | 0 | 0 | 0 | 0 | 1 | 1 | 1 | 2 |  | 15 |
| Heesch (2011) |  | 1 | 1 | 1 | 1 | 1 | 1 | 1 | 1 | 0 |  | 0 | 0 | 1 |  | 1 | 1 | 0 | 0 | 1 | 1 | 1 | 1 | 2 |  | 17 |
| Hurtig-Wennflof (2010) |  | 1 | 1 | 0 | 0 | 1 | 1 | 1 | 1 | 1 |  | 1 | 0 | 1 |  | 1 | 1 | 1 | 1 | 1 | 0 | 1 | 1 | 1 |  | 17 |
| Hooker (2011) |  | 1 | 1 | 1 | 0 | n/a | 1 | 0 | 1 | 0 |  | 0 | 0 | 1 |  | n/a | n/a | n/a | 1 | n/a | n/a | 1 | 1 | 0 |  | 15 |
| Kochersberger (1996) |  | 1 | 1 | 0 | 0 | n/a | 0 | 1 | 1 | 0 |  | 0 | 0 | 1 |  | 0 | n/a | n/a | 1 | 0 | n/a | 0 | 1 | 0 |  | 11 |
| Leaf (1995) |  | 1 | 1 | 1 | 0 | n/a | 1 | 1 | 1 | 0 |  | 0 | 0 | 1 |  | n/a | n/a | n/a | 1 | n/a | n/a | 0 | 1 | 0 |  | 16 |
| Marsh (2007) |  | 1 | 1 | 1 | 0 | n/a | 1 | 0 | 1 | 0 |  | 0 | 0 | 1 |  | n/a | n/a | n/a | 1 | n/a | n/a | 1 | 1 | 0 |  | 16 |
| Morio (1997) |  | 1 | 1 | 0 | 0 | 1 | 1 | 0 | 1 | 1 |  | 0 | 0 | 1 |  | 1 | 1 | 1 | 1 | 0 | 0 | 1 | 1 | 0 |  | 13 |
| O’Brien-Cousins (1996) |  | 1 | 1 | 0 | 0 | 1 | 0 | 0 | 0 | 0 |  | 1 | 0 | 1 |  | 1 | 1 | n/a | 0 | 0 | 1 | 1 | 1 | 0 |  | 11 |
| Pruitt (2008) |  | 1 | 1 | 1 | 1 | 1 | 0 | 1 | 1 | 0 |  | 0 | 0 | 1 |  | 1 | 0 | 1 | 1 | 1 | 1 | 1 | 1 | 2 |  | 17 |
| Resnick (2001) |  | 1 | 1 | 1 | 0 | n/a | 1 | 1 | 1 | 0 |  | 0 | 0 | 1 |  | n/a | n/a | n/a | 1 | n/a | n/a | 1 | 1 | 0 |  | 16 |
| Resnick (2008) |  | 1 | 1 | 0 | 0 | 1 | 1 | 1 | 1 | 0 |  | 0 | 0 | 1 |  | 1 | 1 | n/a | 1 | 1 | 1 | 0 | 1 | 2 |  | 16 |
| Rutgers (1997) |  | 1 | 1 | 0 | 0 | 1 | 1 | 1 | 1 | 0 |  | 0 | 0 | 1 |  | 0 | 0 | 1 | 1 | 1 | 0 | 0 | 1 | 0 |  | 11 |
| Seale (2002) |  | 1 | 1 | 0 | 0 | 1 | 1 | 1 | 1 | 0 |  | 1 | 0 | 1 |  | 1 | 0 | 1 | 0 | 1 | 0 | 1 | 1 | 0 |  | 13 |
| Stel (2004) |  | 1 | 1 | 0 | 0 | 1 | 1 | 1 | 1 | 1 |  | 0 | 0 | 1 |  | 1 | 0 | 0 | 1 | 0 | 0 | 1 | 1 | 2 |  | 14 |
| Storti (2007) |  | 1 | 1 | 1 | 0 | n/a | 1 | 1 | 1 | 0 |  | 0 | 0 | 1 |  | n/a | n/a | n/a | 1 | n/a | n/a | 1 | 1 | 0 |  | 16 |
| Washburn (1993) |  | 1 | 1 | 1 | 0 | 1 | 1 | 1 | 1 | 0 |  | 1 | 0 | 1 |  | 0 | 1 | 1 | 0 | 0 | 1 | 1 | 1 | 2 |  | 16 |
| Washburn (1999) |  | 1 | 1 | 0 | 0 | 1 | 1 | 1 | 1 | 0 |  | 0 | 0 | 1 |  | 0 | 0 | 0 | 0 | 0 | 0 | 1 | 1 | 0 |  | 9 |
| Washburn (1990) |  | 1 | 1 | 1 | 1 | 1 | 1 | 1 | 1 | 1 |  | 0 | 0 | 1 |  | 1 | 0 | n/a | 0 | 1 | 0 | 1 | 1 | 2 |  | 17 |
| Weiss (1990) |  | 1 | 1 | 1 | 0 | 1 | 1 | 0 | 0 | 0 |  | 1 | 0 | 1 |  | 1 | 0 | n/a | 1 | 1 | 0 | 1 | 1 | 2 |  | 15 |
| Yasunago (2007) |  | 1 | 1 | 1 | 0 | 1 | 1 | 0 | 1 | 0 |  | 1 | 0 | 1 |  | 0 | 1 | 0 | 1 | 0 | 1 | 1 | 1 | 2 |  | 15 |
| Zalewski (2009) |  | 1 | 1 | 1 | 1 | 1 | 1 | 1 | 1 | 1 |  | 0 | 0 | 1 |  | 1 | 0 | 0 | 1 | 1 | 0 | 1 | 1 | 2 |  | 17 |

Questions used to evaluate study quality [29]

**Reporting**

1. Is the hypothesis/aim/objective clearly described?
2. Are the operational definitions of the main physical activity constructs to be validated clearly described in the Introduction or Method section
3. Are the characteristics of the participants to be included in the study clearly described?
4. Are the distributions of principle cofounders clearly described?
5. For studies evaluating an existing measure has the original source been cited? For studies evaluating a modified version of an existing measure, has the original source been cited and the modifications described.
6. Are the methods of administration and/or data reduction for the self-report measure and the reference measure clearly described?
7. Have the characteristics of the participants with missing, incomplete, and/or invalid data been described?
8. Does the study provide information about the variability in the data for the main physical activity constructs?
9. Have limits of agreement and/or confidence interval been reported for the main analysis?

**Reporting Score: ___/9**

**External Validity.**

1. Were the individuals asked to participate in the study representative of the entire population from which they were recruited?
2. Were the participants who were enrolled in the study representative of the entire population from which they were recruited?
3. Was the self-report measure administration (e.g., researcher-participant contact, survey mode etc) representative of the procedures applied under epidemiological or behavioral research constraints?

**External Validity Score: ____/3**

**Internal Validity.**

1. Was an attempt made to minimize altered physical activity behaviour by the participant in response to awareness and burden of measurement?
2. Was an attempt made to blind research staff to the activity levels or characteristics of the participants to prevent leading responses to the self-report measure?
3. Does the reference measure assess the physical activity construct(s) of interest with greater accuracy than the self-report measure, and are errors in the reference method uncorrelated with errors in the self-report measure?
4. Did the self-report measure and the reference measure assess physical activity in the same time frame?
5. Was compliance with the measurement protocol acceptable?
6. Was reproducibility of the main physical activity constructs reported for the self-report measure?
7. Were statistical tests used appropriate to assess validity for the main physical activity constructs between the self-report measure and the reference measure?
8. If any of the results of the study were based on ”data dredging” was this made clear?
9. Did the study have sufficient sample size to assess agreement?

**Internal Validity Score: ____/9**

1. Notes: EV = External Validity, IV = Internal Validity, 1 = Yes, 0 = No for all questions except IV question 9, where 2 = Yes, 1 = Partially & 0 = No. When not applicable (n/a) to study, question was given 1 point. [↑](#endnote-ref-2)
