# Supplementary material for: Direct and indirect measurement of physical activity in older adults: a systematic review of the literature
Source: Int J Behav Nutr Phys Act. 2012 Dec 18;9:148. doi: 10.1186/1479-5868-9-148 (PMC3549726; doi:10.1186/1479-5868-9-148)
Supplement: Additional file 3 — Title: Characteristics of studies comparing direct and indirect measures of physical activity in older adults. Description: This document contains a table in which the key details (First author, sample, age (mean (SD), age range), sample size, direct measure (units), indirect measure (units), measurement details (timing, cut-points, epoch lengths, tests, and correlations) of studies comparing direct and indirect measures of physical activity in older adults have been summarized. [file 1479-5868-9-148-S3.docx]

**Additional File 3. Characteristics of Studies Comparing Direct and Indirect Measures of Physical Activity in Older Adults**

| **First Author (Year)** | **Age range or mean (SD)** | **Sample** | **N** | | **M** | **F** | **Direct Measure (units)** | **Indirect Measure (units)** | **Measurement Details (i.e., timing (e.g., recall period, timing of measures in relation to each other), cut-points, epochs)** | **Tests** | **R or Range of R** | |
| --- | --- | --- | --- | --- | --- | --- | --- | --- | --- | --- | --- | --- |
|  |  |  |  |  |  |  |  |  |  |  | **total PA** | **subcategories of PA** |
| Bonnefoy (2001) | 66-82  73.4 (4.1) | Healthy community dwelling men | 19 | 19 | | 0 | Doubly labeled water (kJ·day^-1^) | 1. MLTPAQ (kcal·day^-1^); 2. YPAS (kcal·week^-1^& units/month); 3. MBQ (Points); 4. CAQ (kcal·week^-1^); 5. 7 Day Recall (hours ·week^-1^& kcal·day^-1^); 6. Modified Dalloso (units); 7. LRCQ (4 point score); 8. SUAQ (5 point sore & 6 points score); 9. PASE (points); 10. QAPSE (KJ·day^-1^) | *Timing*: Questionnaires (recall period varied from 7 days to past year) administered on last day of direct measurement (14 days) during a 4 hour interview | Pearson/ Spearman. Percent Agreement calculated for comparable measures. | 0.05 to 0.46/ 0.1 to 0.51 | -0.51 to 0.75/  -0.58 to 0.69 |
| Conn (2000) | 66-90  73.65 (5.90) | Community dwelling ambulatory women | 40 | 0 | | 40 | TriTrac Accelerometer (counts·day^-1^; kcal·day^-1^) | 1. PAI (points·day^-1^); 2. MBQ (points); 3. PASE (points) 4. HPLP episodic exercise (points, kcal/day | *Timing:* Questionnaires (recall period 7 days, past year) completed on 1^st^ day of direct measurement (7-9 days); PAI completed for 5-7 days  *Epochs:* 1 min | Unspecified | 0.24 to 0.49 | -0.06 to 0.05 |
| Dinger (2004) | 75.69 (7.86) | Rural older adults | 56 | 43 | | 13 | Actigraph Accelerometer  (counts ·min^-1^) | PASE (points) | *Timing*: PASE (7 days) completed at end 7 day of direct measurement.  *Epochs:* 1 min | Spearman | 0.43 | 0.11 to 0.38 |
| Gerdhem (2008) | 80.0-80.7 | Subgroup of the OPRA study (original sample was randomly recruited) | 57 | 0 | | 57 | MTI Accelerometer  (counts ·min^-1^;  min·day^-1^ inactivity; min·day^-1^ LPA; min·day^-1^ MVPA) | Activity log (min·day^-1^) | *Timing:* Activity log kept for all 7 days of direct measurement.  *Epochs*: 10 sec  *Cut-points*: <83 counts for inactivity, 83–325 counts for LPA, & >325 counts for MPA | Pearson |  | 0.32 to 0.55 |
| Giles (2009) | Most were 65-74, entire range not provided. | Community dwelling adults from list of volunteers for aging studies | 54 | 20 | | 34 | YAMAX-SW700 Pedometer (Steps·week^-1^) | Modified CHAMPS (frequency·week^-1^ &  MET·min^-^·week^-1^) | *Timing*: CHAMPS (past 4 weeks) mailed out, upon receipt of CHAMPS, 2^nd^ CHAMPS (4 weeks) & pedometer mailed out. Pedometer worn for next 7 days).  *Epoch*: 1 min | Spearman | 0.21 to 0.52 | 0.4 to 0.6 |
| Gill (2008) | 71-89  77 | Community dwelling adults enrolled in exercise programs | 48 | 36 | | 12 | Actigraph Accelerometer (counts ·min^-1^) | Phone-FITT (scores) | Phone-FITT (typical week in last month) administered after 7 days direct measurement | Spearman | 0.56 to 0.57 | 0.29 to 0.46 |
| Hagiwara (2008) | 72.6 (4.9) | Cognitively healthy adults | 325 | 134 | | 191 | Life Corder Accelerometer  (kcal·wt ·day^-1^ &  Steps·day^-1^) | 1. PASE Japanese (Points); 2. JALSPAQ (MET·h^-1^) | *Timing:* Direct measurement (3 days) beginning the day after PASE (7 days) administration. Unclear when JALSPAQ administered | Spearman | -0.02 to 0.38 |  |
| Harada (2001) | 65-89  75(6) | Older adults from retirement homes & community centers. | 87 | 33 | | 54 | 2 Mini Logger (ankle and waist) Accelerometer with HR monitor (counts/6 days) | 1. CHAMPS (kcal·week^-1^) 2. YPAS (kcal·week^-1^) 3. PASE (points) | *Timing*: 1st questionnaire (2 weeks, 4 weeks, past month & typical week) completed at start of direct measurement (6 days), 2^nd^ questionnaire completed during direct measurement, & 3^rd^ questionnaire completed directly after direct measurement. Order of 3 questionnaires administration was random.  *Epochs*: 1 min | Pearson | 0.27 to 0.78 | 0.36  to 0.59 |
| Harris (2009) | 73.6(6.1) | Community dwelling ambulatory adults who were registered with a primary care practice | 234 | 110 | | 124 | 1. Yamax Digi-Walker SW-200 (pedometer; counts·day^-1^; steps·day^-1^) 2. Actigraph Accelerometer (counts·day^-1^; steps·day^-1^) | Modified ZPAQ (kcal· kg ·day^-1^) | *Timing:* Questionnaire (past week, past month, or usual activity). Both direct measures taken over 7 days. Unclear when to measurements were taken compared to each other.  *Epochs*: 5 sec | Pearson | 0.34 to 0.36 |  |
| Heesch (2011) | 65-89  72.6(5.9) | Community dwelling, English speaking, ambulatory, cognitively intact older adults | 53 | 27 | | 26 | Yamax Digi-Walker SW-200 (pedometer; counts·day^-1^) | AAS ( min·day^-1^ PA, min·day^-1^ MVPA) | *Timing:* Questionnaire (past week) administered after 7 days of direct measurement + step log | Spearman | 0.42 | 0.31 to 0.42 |
| Hurtig-Wennflof (2010) | 66-91 | Retired independently living older adults | 54 | 31 | | 23 | Actigraph Accelerometer (min·day^-1^ sitting, min·day^-1^ in mixed MPA, min·day^-1^ in ambulatory MPA, min·day^-1^ in VPA, counts ·min^-1^) | Swedish translation of the IPAQ, modified for the elderly | *Timing:* Questionnaire (past week) administered after 7 days of direct measurement.  *Epochs*: 15 sec.  *Cut-points:* Sitting < 100 counts/min, moderate activity = 760–2019 counts/min, moderate activity (ambulatory activities) = 2020–4944 counts/min and vigorous activity = >4944 counts/min | Spearman |  | -0.35 to 0.47 |
| Morio (1997) | 70.1(2.7) | Healthy elderly adults | 12 | 6 | | 6 | 1. Doubly labeled water (MJ·day^-1^) 2. HR monitoring (MJ·day^-1^) | Factorial method/activity log (MJ·day^-1^) | *Timing:* Daily energy expenditure measured for 3 days using calorimeters. Then in free living conditions, doubly labeled water was measured for 17 days, while activity was recorded in a log for 14 days. HR was recorded minute by minute on 4 randomly chosen days in the study period. | Bland-Altman method used to examine agreement | n/a | n/a |
| Pruitt (2008) | 70-86  77.6 (4.0) | Community dwelling sedentary older adults at risk for mobility disability; not cognitively impaired | 106 | 71 | | 35 | Accelerometer (counts·hr^-1^; min·day^-1^ above thresh_IND_; counts·day^-1^ above thresh_IND_;  # bouts·day^-1^ above thresh_IND_) | CHAMPS (kcal·week^-1^; frequency/week; MET·hr·week ^-1^) | *Timing:* Questionnaire (past 4 wks.) administered at 6 or 12 mos. of an intervention. Unclear when questionnaire administered compared to direct measure (7 days).  *Epochs*: 1 min  Cut-points: Average counts/min during a 400-m Activity counts above this level were operationally defined as meaningful activity. | Spearman | 0.08 to 0.42 | 0.03 to 0.31 |
| Rutgers (1997) | 73(3)  68-78 | Healthy weight stable community dwelling volunteers | 13 | 0 | | 13 | 1. Heart rate monitoring (kcal/min) 2. Indirect calorimetry (kcal/min) | 24 hr recall (kcal/min) | *Timing:* Individual calibration curves & group calibration curves were calculated; 3 days of minute by minute heart rate monitoring within 2 weeks; 24 hour activity recall at the end of each day. |  | n/a | n/a |
| Seale (2002) | 74 | Rural Elderly | 27 | 14 | | 13 | Doubly labeled water (MJ·day^-1^) | 7 day recall (MJ·day^-1^) | *Timing:* Indirect calorimetry on first day. Doubly labeled water for 6 consecutive days and diet records for 3 consecutive days over 2 weeks. 7 day recall administered on two occasions during the two weeks |  | n/a | n/a |
| Stel (2004) | 69-92 | Subsample of LASA who fell in last year and a random sample of adults who did not fall during previous year | 439 | ? | | ? | DIGI Walker Yamax Pedometer (steps·day^-1^) | 1. LAPAQ (min· 2 weeks^-1^) 2. 7 day diary (min· 2 weeks^-1^) | *Timing:* Questionnaire (2 weeks) administered followed by direct measurement (7 days); Diary kept for entire period of direct measurement (7 days) | Spearman | 0.43 to 0.56 |  |
| Washburn (1990) | 73  65-91 | Community-dwelling volunteers | 103 | 52 | | 51 | Caltrac Accelerometer (units unclear) | 1. Activity diary (min·day^-1^) 2. BRFSS (min·day^-1^) | *Timing:* Within 1 week of interview, activity diary was completed for 3 days | Spearman | 0.12 to 0.29 |  |
| Washburn (1993) | 67-80 | Healthy volunteers | 193 | ? | | ? | Caltrac Accelerometer (counts/day) | 1. PASE (points) 2. Activity diary (mets·day^-1^) 3. Global self-reported activity item | *Timing:* Diary and direct measurement was completed for 3 days. On third day global self-assessment was completed | Unspecified | 0.31 to 0.79 |  |
| Washburn (1999) | 67-80 | Healthy volunteers | 20 | 3 | | 17 | Computer Science and Applications (CSA) Inc, Portable Accelerometer  (counts·5 min^-1^) | PASE (points) | *Timing*: Questionnaire (7 days) completed following direct measurement (3 days).  *Epochs*: 5 min | Spearman | 0.11 to 0.64 | -0.06 to 0.66 |
| Yasunago (2007) | 65-99 | Elderly adults who institutionalized and/or hospitalized | 147 | 61 | | 86 | Accelerometer  (steps·day^-1^; min ·day^-1^) | PAQ-EJ (MET·hr·week ^-1^) | *Timing:* Direct measurement (1 month) followed by questionnaire (1 week) completion. | Spearman | 0.41 | 0.28 to 0.53 |
| Zalewski (2009) | 83.8 | Adults from independent or assisted living apartments | 59 | 14 | | 45 | Accelerometer  (steps·day^-1^) | PASE (points) | *Timing:* Questionnaire (7 days) administered before direct measurement (7 days) | Pearson | 0.07 |  |
