# Supplementary material for: Direct and indirect measurement of physical activity in older adults: a systematic review of the literature
Source: Int J Behav Nutr Phys Act. 2012 Dec 18;9:148. doi: 10.1186/1479-5868-9-148 (PMC3549726; doi:10.1186/1479-5868-9-148)
Supplement: Additional file 4 — Characteristics of studies comparing indirect measures of physical activity in older adults. Description: This document contains a table in which the key details (First author, sample, age (mean (SD), age range), sample size, direct measure (units), indirect measure (units), measurement details (timing, cut-points, epoch lengths, tests, and correlations) of studies comparing indirect measures of physical activity with other indirect measures of physical activity in older adults have been summarized. [file 1479-5868-9-148-S4.docx]

**Additional File 4. Characteristics of studies comparing indirect measures of physical activity in older adults**

| **First Author^[[1]](#footnote-1)^ (Year)** | **Age range (yrs) or Mean Age (SD)** | **Sample** | **N** | **M** | **F** | **Measures (Units)** | **Measurement Details (i.e., timing (e.g., recall period, timing of measures in relation to each other), cut-points, epochs)** | **Test** | **R or Range of R** |  |
| --- | --- | --- | --- | --- | --- | --- | --- | --- | --- | --- |
|  |  |  |  |  |  |  |  |  | **Total PA** | **Subcategories of PA** |
| Bonnefoy (2001) | 66-82  73.4 (4.1) | Healthy community dwelling men | 19 | 19 | 0 | 1. MLTPAQ (kcal·day^-1^); 2. YPAS (kcal·week^-1^& units/month); 3. MBQ (Points); 4. CAQ (kcal·week^-1^); 5. 7 Day Recall (hours ·week^-1^& kcal·day^-1^); 6. Modified Dalloso (units); 7. LRCQ (4 point score); 8. SUAQ (5 point sore & 6 points score); 9. PASE (points); 10. QAPSE (KJ·day^-1^) | *Timing*: Questionnaires (recall period varied from 7 days to past year) administered on last day of direct measurement (14 days) during a 4 hour interview | Agreement between comparable questionnaires was not examined, but the authors included necessary data to perform the calculation | n/a | n/a |
| Conn (2000) | 66-90  73.65 (5.90) | Community dwelling ambulatory women | 40 | 0 | 40 | 1. PAI (points·day^-1^); 2. MBQ (points); 3. PASE (points) 4. HPLP episodic exercise (points, kcal/day) | *Timing:* Questionnaires (recall period 7 days, past year) completed on 1^st^ day of direct measurement (7-9 days); PAI completed for 5-7 days  *Epochs:* 1 min | Unspecified | 0.4-0.59 | 0.12-0.25 |
| Dominques-Berjon (1999) | One age group > 65 | Sample of adults who answered the BHIS in 1992, study looked at age groups including 1 > 65 | ? | ? | ? | 1. Questions from the Welsh Heart Health Survey (Times/week) 2. Exercise Induced Sweating from BHIS (times/week) | *Timing*: Both PA measure collected during the BHIS survey in 1992 (telephone) | Weighted cohen κ coefficient for assessment of agreement | n/a | n/a |
| Hagiwara (2008) | 72.6 (4.9) | Cognitively healthy adults | 325 | 134 | 191 | 1. PASE Japanese (Points); 2. JALSPAQ (MET·h^-1^) | *Timing:* Direct measurement (3 days) beginning the day after PASE (7 days) administration. Unclear when JALSPAQ administered | Spearman | 0.47-0.48 |  |
| Harada (2001) | 65-89  75(6) | Older adults from retirement homes & community centers. | 87 | 33 | 54 | 1. CHAMPS (kcal·week^-1^) 2. YPAS (kcal·week^-1^) 3. PASE (points) | *Timing*: 1st questionnaire (2 weeks, 4 weeks, past month & typical week) completed at start of direct measurement (6 days), 2^nd^ questionnaire completed during direct measurement, & 3^rd^ questionnaire completed directly after direct measurement. Order of 3 questionnaires administration was random.  *Epochs*: 1 min | Pearson. Agreement between comparable questionnaires was not examined, but the authors included necessary data to perform the calculation. | 0.61-0.64 | 0.18-0.68 |
| O’Brien-Cousins (1996) | 70-98  77 | Community dwelling women aged 70 years and over, volunteers from randomly selected older adults facilities residences | ? | 0 | ? | 1. OA-ESI (kcal·week^-1^) 2. Lifelong PA from Godin (1987) 3. Exercise induced sweating question from (Godin & Shepard, 1982) | *Timing:* Mail out survey including OA-ESI and the additional PA questions | Pearson | 0.41-0.49 |  |
| Resnick (2008) –SENIORS STUDY | 76.2 (6.2) | Community-dwelling | 150 | ? Approx 30% | Approx. 70% | 1. YPAS (hrs·week^-1^; kcal·week^-1^) 2. CHAMPS (hrs·week^-1^; kcal·week^-1^) | *Timing:* YPAS and CHAMPS administered at 6 months | Pearson. Agreement between comparable questionnaires was not examined, but the authors included necessary data to perform the calculation. | 0.47-0.66 |  |
| Resnick (2008) HIP study | 80.7 (6.8) | Sustained hip fracture within 15 days of recruitment | 150 | ? | ? | 1. YPAS (hrs·week^-1^; kcal·week^-1^) 2. CHAMPS (hrs·week^-1^; kcal·week^-1^) | *Timing:* YPAS administered at baseline, and YPAS and CHAMPS 6 months, 12 months, | Pearson. Agreement between comparable questionnaires was not examined, but the authors included | 0.34-0.43 |  |
| Stel (2004) | 69-92 | Subsample of LASA who fell in last year and a random sample of adults who did not fall during previous year | 439 | ? | ? | 1. LAPAQ (min· 2 weeks^-1^) 2. 7 day diary (min· 2 weeks^-1^) | *Timing:* Questionnaire (2 weeks) administered followed by direct measurement (7days); Diary kept for entire period of direct measurement (7 days) | Spearman. Agreement was assessed using the Bland Altman method | 0.68 | 0.5-0.84 |
| Washburn (1990) | 73  65-91 | Community-dwelling volunteers | 103 | 52 | 51 | 1. Activity diary (min·day^-1^) 2. BRFSS (min·day^-1^) | *Timing:* Within 1 week of interview, activity diary was completed for 3 days | Assessed agreement using absolute and average reporting error. | n/a | n/a |
| Washburn (1993) | 67-80 | Healthy volunteers | 193 | ? | ? | 1. PASE (points) 2. Activity diary (mets·day^-1^)\ 3. Global self-reported activity item | *Timing:* Diary was completed for 3 days. On third day global self-assessment was completed | Unspecified | 0.44-0.85 |  |
| Wiess (1990)^[[2]](#endnote-1)^ | ? | US population | ? | ? | ? | 1. Individual EE (kcal.kg/day) from the HD/PD Supplement to the 1985 NHIS 2. Job related activity item 3. Main daily activity 4. Activity compared to peers (3 levels) 5. Activity compared to peers (5 levels) | *Timing:* Telephone interview all items asking about frequency of activity in last two weeks | Spearman. | 0.15-0.32 |  |

1. [↑](#footnote-ref-1)
2. Weiss (1990) reports on the data from a national survey looking at adult health and use of medical services. Description of overall sample size, gender distribution, and mean standard deviations, and ranges in the older adults groups (65-74 years, 75+) not provided [↑](#endnote-ref-1)
