# Supplementary material for: Direct and indirect measurement of physical activity in older adults: a systematic review of the literature
Source: Int J Behav Nutr Phys Act. 2012 Dec 18;9:148. doi: 10.1186/1479-5868-9-148 (PMC3549726; doi:10.1186/1479-5868-9-148)
Supplement: Additional file 5 — Characteristics of studies comparing direct measures of physical activity in older adults. Description: This document contains a table in which the key details (First author, sample, age (mean (SD), age range), sample size, direct measure (units), indirect measure (units), measurement details (timing, cut-points, epoch lengths, tests, and correlations) of studies comparing direct measures of physical activity with other direct measures of physical activity in older adults have been summarized. [file 1479-5868-9-148-S5.docx]

**Additional File 5. Characteristics of studies comparing direct measures of physical activity in older adults**

| **First Author (Year)** | **Age range or mean (SD)** | **Sample** | **N** | **M** | **F** | **Measures (Units)** | **Measurement Details (i.e., timing of measures in relation to each other), setting, cut-points, epochs)** | **Test** | **R or Range of R** |
| --- | --- | --- | --- | --- | --- | --- | --- | --- | --- |
| Ayabe (2008) part 2^[[1]](#endnote-1)^ | 69(4) | Active and inactive older adults | 28^[[2]](#endnote-2)^ | 13 | 15 | 1. Life Corder (pedometer; steps/day). 2. EC-200 (Pedometer; steps/day | *Timing:* Both measures for 7 days  *Setting:* real world.  *Epochs:* 4 second for Life Corder.  *Cut-points:* 10 categories of PA inactive, 1.8, 2.3, 2.9, 3.6, 4.3, 5.2, 6.1, 7.1 and .8.3 METs. Categories 1–3, 4–6 and 7–9 were defined as light (< METs), moderate (3–6 METs) and vigorous-intensity (>6 METs) | Pearson. Bland Altman method used to examine agreement. | 0.69-0.97 |
| Bergman (2008) Study 1 | 78.6(13.1) | Older adults residing in assisted living community | 21 | 5 | 16 | 1. StepWatch 3 Step Activity Monitor (pedometer; steps) 2. Yamax Digi-Walker SW-200 (pedometer; steps) | *Timing*: Both measures compared over 161 meters.  *Setting*: Laboratory/controlled setting | Pearson. Bland Altman method used to examine agreement. | -0.28 to 0.99 |
| Bergman (2008) Study 2 | 76.7(16.0) | Older adults residing in assisted living community | 13 | 4 | 9 | 1. StepWatch 3 Step Activity Monitor (pedometer; hours worn/day; steps/day) 2. Yamax Digi-Walker SW-200 (pedometer; hours worn/day, steps/day) | *Timing*: Both measures worn for 7 days  Setting: real world | T-test used to examine difference in step count between two pedometers | n/a |
| Cyarto (2004) | 79.4 (8.2) nursing home  70.6(5.5) senior centres | Adults from nursing home & senior centres | 54 | 10 | 44 | 1. Yamax Digi-Walker SW-200 (pedometer; steps/trial) 2. Direct observation (steps/trial) | *Timing*: Three 13m walk trials on treadmill at slow, medium and fast self-paced walking speeds  *Setting:* Laboratory/controlled setting | Percent error/agreement | n/a |
| Fehling (1999) | 70.6(3.7) | Healthy volunteers from STOP-IT (exercise study) | 86 | 44 | 42 | 1. Caltrac (accelerometer, kcal/min) 2. Tritrac (accelerometer, kcal/min) 3. Indirect calorimetry (kcal/min | *Timing*: Both measures worn during submaximal treadmill & stepping tests. Metabolic measurements from last 3 min of treadmill walking & last 2 minutes of stepping.  *Setting*: Laboratory/controlled setting | Repeated measures ANOVA | n/a |
| Grant (2008) | 65-87 | Volunteers from exercise classes | 21 | 10 | 11 | 1. Active Pal (accelerometer; total steps; steps·min^-1^) 2. New-Lifestyles Digi-Walker SW-200 (pedometer; total steps; steps·min^-1^) 3. New-Lifestyles NL-200 (pedometer; total steps; steps·min^-1^) 4. Direct observation (recorded on camcorder) | *Timing*: All measures taken during walked trials on treadmill at 5 different speeds & during walk outside on 500m course  *Setting*: Laboratory/controlled setting | Bland Altman method used to examine agreement. | n/a |
| Harris (2009) | 73.6(6.1) | Community dwelling ambulatory adults who were registered with a primary care practice | 234 | 110 | 124 | 1. Yamax Digi-Walker SW-200 (pedometer; counts·day^-1^; steps·day^-1^) 2. Actigraph Accelerometer (counts·day^-1^; steps·day^-1^) | *Timing:* Questionnaire (past week, past month, or usual activity). Both direct measures taken over 7 days. Unclear when to measurements were taken compared to each other.  *Epochs*: 5 sec | Pearson. | 0.82-0.86 |
| Hooker (2011) | 74(6)  65-87 | Older adults of varying body composition | 23 | 12 | 11 | 1. Indirect calorimetry (kcal/kg/min 2. Actical accelerometer (counts/min; /kcal/kg/min) | Timing: resting, sitting, household cleaning, and locomotion measured using accelerometer and portable metabolic measurement system  Setting: Laboratory/controlled setting  *Epochs:* 1 minute epochs  *Cut-points:* Developed 1 overall AC cut-point of 1065 for all 3 groups (obese and non-obese adults, older adults) and 3 group-specific activity count cut-points (1107, 1634, and 431) for division between light and moderate physical activity | Regression Analysis | 0.92 |
| Kochersberger (1996)^[[3]](#endnote-3)^ | 76 | Residents of a nursing home | 8 | ? | ? | 1. Actigraph (accelerometer; counts·min^-1^) 2. Tritrac (accelerometer; counts·min^-1^) | *Timing:* Both measure taken for 5 minutes of sitting, and 5 minutes of treadmill walking at 1mph and 2mph.  *Setting*: Laboratory/controlled setting  *Epochs*: 1 min  *Cut-points:* 20 activity counts per minute cutoff for sitting | Pearson | 0.77 |
| Leaf (1995) | 71 | Healthy community dwelling older adults | 20 | 5 | 15 | 1. Caltrac (accelerometer; kcal predicted from acceleration in vertical plane) 2. Indirect calorimetry (kcal) 3. ACSM predictions of kcal for walking | *Timing:* Both measures taken during a 10 minute treadmill walking test  *Setting*: Laboratory/controlled setting | Unspecified correlation coefficient. Multiple regression also conducted. | 0.33 |
| Marsh (2007) | 75.8(4.2) | Individuals at risk of mobility disability | 29 | 9 | 20 | 1. Accusplit Eagle 120 (pedometer; total steps) 2. NL-2000 (pedometer; total steps) 3. IDEEA pattern recognition device (total steps) 4. Direct observation (average total steps by two observers) | *Timing:* All devices worn during a walk at preferred speed around 1 131m indoor track.  Setting: Laboratory/controlled setting | Spearman. Bland Altman method used to examine agreement | 0.51-0.98 |
| Morio (1997) | 70.1(2.7) | Healthy elderly adults | 12 | 6 | 6 | 1. Doubly labeled water (MJ·day^-1^) 2. HR monitoring (MJ·day^-1^) | *Timing:* Daily energy expenditure measured for 3 days using calorimeters. Then in free-living conditions, doubly labeled water was measured for 17 days, while activity was recorded in a log for 14 days. HR was recorded minute by minute on 4 randomly chosen days in the study period.  Setting: Real world | Bland-Altman method used to examine agreement | n/a |
| Resnick (2001) | 86(6.1) | Continuing care retirement community | 30 | 22 | 8 | 1. SAM 2. Direct observation (average of steps by two observers) | Timing: Both measures taken over 2 one minute walk trials at preferred speed  Setting: Laboratory/controlled setting | Unspecified correlation. Percent error/agreement | 0.97-0.98 |
| Rutgers (1997) | 73(3)  68-78 | Healthy weight stable community dwelling volunteers | 13 | 0 | 13 | 1. Heart rate monitoring (kcal/min) 2. Indirect calorimetry (kcal/min) | Timing: Individual calibration curves & group calibration curves were calculated; 3 days of minute by minute heart rate monitoring within 2 weeks; 24 hour activity recall at the end of each day.  Setting: Real world | Pearson | 0.37 |
| Storti (2007) | 79.2 (6.0) | Community dwelling older adults | 34 | 10 | 24 | 1. Yamax Digi-Walker SW-200 (pedometer; steps) 2. Actigraph (accelerometer; steps) 3. SAM (activity monitor; steps) 4. Direct observation (steps) | Timing: All measured taken during a 100m self-paced walk on level surface in straight line  Setting: Laboratory/controlled setting | Percent error/agreement | n/a |

1. Part 1 of study was a calibration exercise on a treadmill in 7 young men. [↑](#endnote-ref-1)
2. Study also examined step counts in younger adults (N=17). Only results specific to the older adult sample (N=28) are presented. [↑](#endnote-ref-2)
3. One of 6 studies that is very briefly described. Mean age of entire sample of nursing home (n=40) residents is 76. [↑](#endnote-ref-3)
